# Chemogenetic activation of locus coeruleus neurons ameliorates the severity of multiple sclerosis

**DOI:** 10.1186/s12974-023-02865-z

**Published:** 2023-09-01

**Authors:** Alejandro Torrillas-de la Cal, Sonia Torres-Sanchez, Lidia Bravo, Meritxell Llorca-Torralba, Jose Antonio Garcia-Partida, Ana I. Arroba, Esther Berrocoso

**Affiliations:** 1 Neuropsychopharmacology and Psychobiology Research Group, Department of Neuroscience, University of Cádiz, 11003 Cádiz, Spain; 2 Ciber de Salud Mental (CIBERSAM), Instituto de Salud Carlos III, 28029 Madrid, Spain; 3 Instituto de Investigación e Innovación Biomédica de Cádiz (INiBICA), Hospital Universitario Puerta del Mar, 11009 Cádiz, Spain; 4 Neuropsychopharmacology and Psychobiology Research Group, Department of Cell Biology and Histology, University of Cádiz, 11003 Cádiz, Spain; 5 Department of Biomedicine, Biotechnology and Public Health (Immunology Area), University of Cádiz, 11003 Cádiz, Spain

**Keywords:** Multiple sclerosis, Experimental autoimmune encephalomyelitis, Locus coeruleus, Noradrenaline, Spinal cord

## Abstract

**Background:**

Most current disease-modifying therapies approved for multiple sclerosis (MS) are immunomodulatory drugs that counteract the aberrant activity of the immune system. Hence, new pharmacological interventions that drive anti-inflammatory activity and neuroprotection would represent interesting alternative therapeutic approaches or complementary strategies to treat progressive forms of MS. There is evidence of reduced noradrenaline levels and alterations to locus coeruleus (LC) noradrenergic neurons in MS patients, as well as in animal models of this disease, potentially factors contributing to the pathophysiology. Drugs that enhance noradrenaline appear to have some beneficial effects in MS, suggesting their potential to dampen the underlying pathology and disease progression.

**Methods:**

Therefore, we explored the consequences of chronic LC noradrenergic neurons activation by chemogenetics in experimental autoimmune encephalomyelitis (EAE) mice, the most widely used experimental model of MS. LC activation from the onset or the peak of motor symptoms was explored as two different therapeutic approaches, assessing the motor and non-motor behavioral changes as EAE progresses, and studying demyelination, inflammation and glial activation in the spinal cord and cerebral cortex during the chronic phase of EAE.

**Results:**

LC activation from the onset of motor symptoms markedly alleviated the motor deficits in EAE mice, as well as their anxiety-like behavior and sickness, in conjunction with reduced demyelination and perivascular infiltration in the spinal cord and glial activation in the spinal cord and prefrontal cortex (PFC). When animals exhibited severe paralysis, LC activation produced a modest alleviation of EAE motor symptoms and it enhanced animal well-being, in association with an improvement of the EAE pathology at the spinal cord and PFC level. Interestingly, the reduced dopamine beta-hydroxylase expression associated with EAE in the spinal cord and PFC was reversed through chemogenetic LC activation.

**Conclusion:**

Therefore, clear anti-inflammatory and neuroprotective effects were produced by the selective activation of LC noradrenergic neurons in EAE mice, having greater benefits when LC activation commenced earlier. Overall, these data suggest noradrenergic LC neurons may be targets to potentially alleviate some of the motor and non-motor symptoms in MS.

**Supplementary Information:**

The online version contains supplementary material available at 10.1186/s12974-023-02865-z.

## Introduction

Multiple sclerosis (MS) is a chronic neurological autoimmune disease that is estimated to affect 2.8 million people worldwide and it represents the leading cause of non-traumatic disability in young adults [1–3]. This pathology affects the central nervous system (CNS) and it produces progressive irreversible demyelination, with severe motor and non-motor consequences [4]. While most current disease-modifying therapies approved for MS are immunomodulatory drugs, mainly aimed at decreasing the frequency of relapses, they have a limited efficacy in halting disease progression [5]. Hence, other pharmacological interventions that effectively drive anti-inflammatory and neuroprotective effects would represent an alternative or additional therapeutic approach to treat MS.

There is evidence that disruptions to physiological noradrenaline homeostasis or signaling might contribute to MS. Indeed, impaired noradrenergic functional connectivity and axonal damage in the locus coeruleus (LC), the main source of noradrenaline in the CNS, has been reported in MS patients [6–8]. Significant astroglial activation in and around the LC, as well as a reduction in LC noradrenaline levels, have also been reported in human post-mortem brains [9]. In the experimental autoimmune encephalomyelitis (EAE) mice model of MS, neuronal damage in the LC and an associated reduction in cortical and spinal cord noradrenaline levels have also been observed [9], reflecting the disease-induced neuronal harm in the LC. Moreover, damage to noradrenergic LC neurons appears to exacerbate the symptomatology in this animal model [10] although not in others [11–13]. These findings clearly point to altered LC physiology in MS that specifically affects noradrenergic neurotransmission. Interestingly, studies in vitro and in vivo have demonstrated that noradrenaline restricts the development of neuroinflammatory activation in the CNS, providing neurotrophic support to neurons and protecting astrocytes, microglia and neurons against oxidative stress [14–17]. Furthermore, central noradrenaline acts as a classic neurotransmitter, critically regulating arousal, attention, anxiety and pain [18–21], relevant non-motor behaviors affected in MS.

Interestingly, as there is no clear loss of LC neurons in MS, as in other neurodegenerative diseases like Alzheimer’s or Parkinson’s disease [22–25], promoting the activity of the LC noradrenergic system might be a therapeutic option in MS. However, the consequences of enhancing the availability of endogenous noradrenaline remain unclear. For example, administering atomoxetine (a noradrenaline reuptake inhibitor) to EAE mice that are already ill does not promote recovery [10], although when administered along with the synthetic noradrenergic precursor L-threo-3,4-dihydroxyphenylserine (L-DOPS), clinical improvements in EAE mice were evident [10]. In MS patients, treatment with noradrenaline reuptake inhibitors (lofepramine or maprotiline) combined with levodopa (which after conversion to dopamine metabolizes to noradrenaline) have been reported to have beneficial effects. However, as dopamine itself exerts beneficial effects on the disease [26], the rise in central noradrenaline levels may not drive these effects. Furthermore, the combination of lofepramine and L-phenylalanine (a precursor of noradrenaline) was initially shown to dampen the clinical symptoms of MS [27], although subsequent studies raised doubts about this therapeutic effect [28]. Therefore, it remains unclear whether limiting neuron damage in the LC or enhancing LC neuronal activity might combat the progression of MS. Indeed, such a therapeutic approach might fail to address the peripheral cardiovascular noradrenergic effects or even peripheral pro-inflammatory actions [29, 30].

Chemogenetic tools, like designer receptors exclusively activated by designer drugs (DREADD), can be used to specifically modulate the activity of LC noradrenergic neurons [31]. Hence, we have explored whether chronic chemogenetic activation of LC noradrenergic neurons has a beneficial effect on the clinical course of EAE, evaluating motor and non-motor behavioral changes as EAE progresses. Furthermore, we studied the effects of this manipulation on demyelination, inflammation and glial activation in the spinal cord and cerebral cortex in the chronic phase of the EAE model. To evaluate the ability of this therapeutic strategy to prevent or restore the alterations induced in the EAE model, chronic chemogenetic activation was first induced from the onset of the disease or at the peak of the motor symptoms, respectively.

## Materials and methods

A more detailed description of the experimental procedures is provided in the Additional file 1.

### Animals, experimental design and groups

**Fig. 1:**
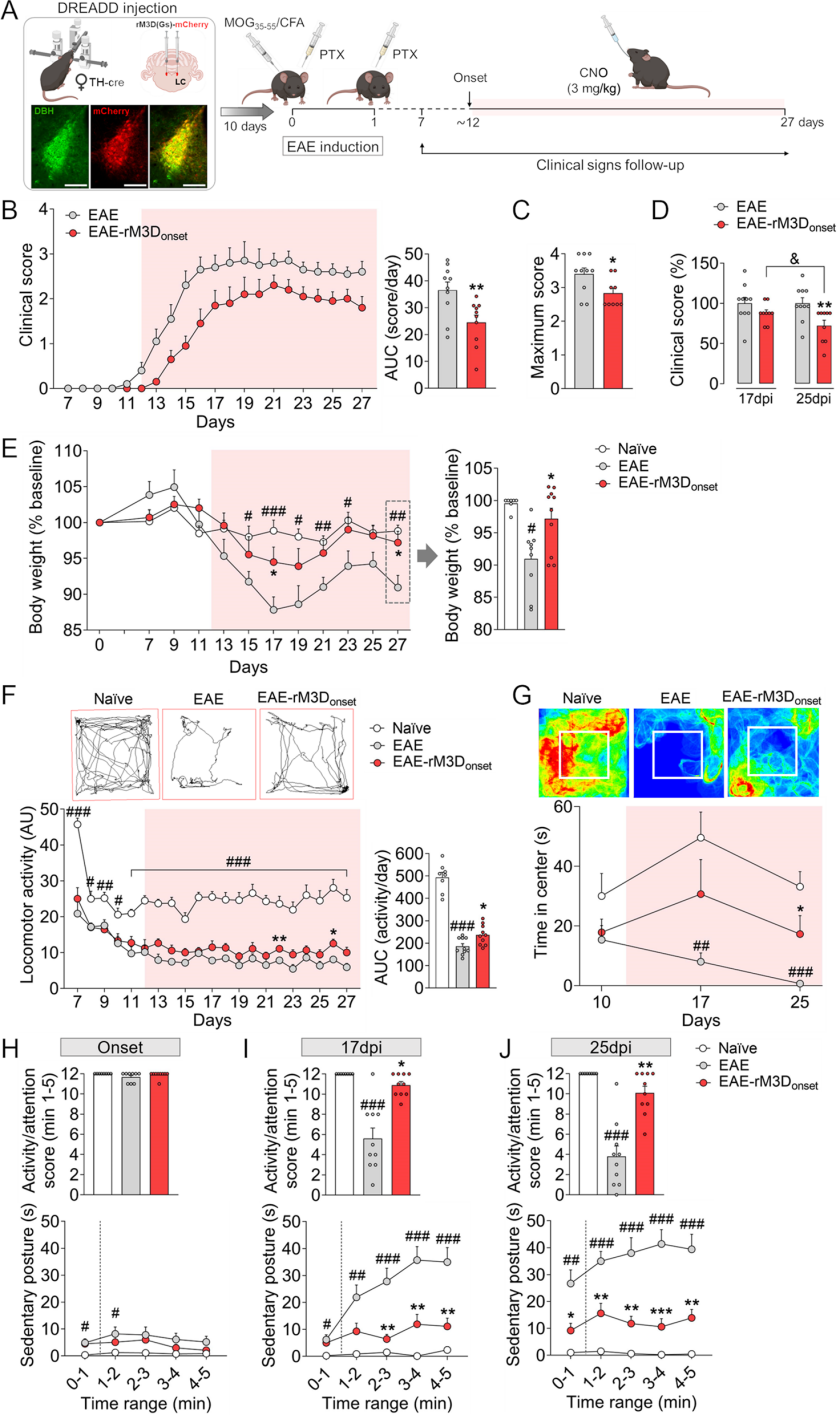
Chronic LC activation from the onset of motor symptoms affects EAE-induced behavioral changes. **A** Experimental timeline showing the design of the study to assess the effect of chronic LC activation from the onset of motor symptoms in EAE. Representative images showing mCherry (red) expression in DBH (green) in the LC. Scale bars: 100 µm. **B** Clinical score recorded daily through the course of EAE and their area under the curve (AUC) from the onset of motor deficit and CNO administration (shaded area). **C** The severity of EAE is expressed as the maximum clinical score achieved and **D** the percentage change relative to the 17 dpi EAE group, for both the peak (17 dpi) and the chronic (25 dpi) phases of EAE. **E** Body weight over the course of EAE expressed as the relative change from baseline (at the beginning of experiments) and that at the end of experiments (27 dpi). **F** Representative activity traces (chronic phase) and the spontaneous locomotor activity expressed as the total distance travelled (arbitrary units, AU), and the AUC from the onset of motor deficit and CNO administration (shaded area). **G** Representative heat maps (chronic phase) and the relative time spent in the central area of the arena before the onset of motor deficit and CNO administration (10 dpi), and at the peak (17 dpi) and chronic (25 dpi) phases of EAE. **H**–**J** Activity/attention score (above) and time spent in the sedentary posture (below) at **H** the onset, **I** and at the peak (17 dpi) and **J** in the chronic (25 dpi) phase of EAE. The data represent the mean + SEM and each point corresponds to an individual mouse (*n* = 8–10 per group). ^#^p < 0.05, ^##^p < 0.01, ^###^p < 0.001 EAE versus naïve; *p < 0.05, **p < 0.01, ***p < 0.001 EAE-rM3D_onset_ versus EAE; ^&^*p* < 0.05 EAE-rM3D_onset_ 25 dpi versus 17 dpi (Additional file 1: Table S2). Some elements of this figure were created with BioRender.com

Female C57BL/6 J TH:Cre mice were used in these studies and maintained under standard laboratory conditions at the University of Cadiz. The TH:Cre founders were provided by INFRAFRONTIER/EMMA (EM:00254) [32]. The Cre-dependent DREADD virus was injected bilaterally into the LC and 10 days later, EAE was induced and the clinical symptoms were monitored from day 7 post-induction (dpi). For chronic chemogenetic LC activation, clozapine-N-oxide (CNO, 3 mg/kg) was administered orally on a daily basis from the onset (~ 12 dpi) or the peak of motor symptoms (peak phase of EAE: clinical score ≥ 3, ~ 17 dpi). The animals were then killed in the chronic phase of EAE (from ~ 20 dpi), collecting the spinal cord and brain tissues at 27–29 dpi to assess the neurobiological alterations (Figs. 1A, 5A). The experimental groups established in this study were control animals (naïve group), and groups of EAE animals administered a control-DREADD or rM3D(Gs)-DREADD into the LC (EAE and EAE-rM3D groups, respectively) and treated chronically with CNO or the vehicle alone.

### DREADD virus injection

Mice were injected with a DREADD virus (rM3D(Gs)-DREADD or control-DREADD) bilaterally into the LC (AP − 5.3 mm, ML ± 1 mm, − 3.6 mm from the surface of the skull) [33] and DREADD expression in the LC was verified at the end of the experiments (Additional file 1: Fig. S1).

### EAE induction and evaluation

Mice were immunized by subcutaneous injection of mouse myelin oligodendrocyte glycoprotein peptide (MOG_35–55_) in complete Freund’s adjuvant (CFA), and pertussis toxin (PTX) was then administered intraperitoneally, 2 and 24 h after immunization (Hooke Laboratories Inc, USA). Clinical signs of EAE were assessed daily obtaining a clinical score of 0–4.5. The area under the curve (AUC) values of this score, the maximum score achieved and the relative change in the clinical score at the peak (17 dpi) and in the chronic (25 dpi) phase of EAE (relativized to EAE group at the peak phase) were also analyzed. Body weight was also monitored every 2 days.

### Open field test

Mice were placed individually in a square arena (45 × 45 × 35 cm) for 5 min to allow them to explore the space freely. Spontaneous locomotor activity was measured as the total distance travelled, expressed in arbitrary units (AU), as well as the AUC values of the total distance travelled. The time spent in the central area of the arena was also measured as a readout for anxiety-like behavior. To evaluate “sickness behavior”, the time spent in a specific sedentary posture and the activity/attention score were also assessed [34].

### Histological and immunohistochemical assays

Hematoxylin–eosin staining was performed on the spinal cord to evaluate the degree of perivascular infiltration [35, 36]. Immunohistochemistry and immunofluorescence assays were carried out to assess the expression of tyrosine hydroxylase (TH) and glial fibrillary acidic protein (GFAP) in the LC, and the co-expression of mCherry (the DREADD reporter protein) and dopamine beta-hydroxylase (DBH) in the LC, A5 and A7. In addition, the expression of myelin basic protein (MBP), GFAP, ionized calcium-binding adapter molecule 1 (Iba1), arginase-1 (Arg1), inducible nitric oxide synthase (iNOS) and DBH were evaluated in the spinal cord, the prefrontal cortex (PFC): prelimbic (PL) and infralimbic (IL) and motor cortices: secondary (M2) and primary (M1). Detailed information about antibodies used is included in Additional file 1: Table S1. Images were acquired on an Olympus BX60 microscope (Olympus, Spain) or a confocal Zeiss LSM 880 (Carl Zeiss Microscopy GmbH, Germany) and analyzed with Fiji Imaging software (USA).

### Statistical analysis

The data are represented as the means + standard error of the mean (SEM) and the results were analyzed with a Student’s t-test, one- or two-way, or repeated measures ANOVA, followed by Bonferroni or Dunnett’s post hoc tests. For non-parametric data, Mann–Whitney *U* or Kruskal–Wallis tests were applied, followed by a Dunn’s post hoc test. Correlations were assessed using Pearson or the non-parametric Spearman correlation coefficients. Significance was accepted at *p* < 0.05 (see Additional file 1: Tables S2–S11 for more details on the statistical analysis).

## Results

### EAE-induced changes in the LC

The possible alterations to the LC induced by EAE were first assessed and a significant increase in the area occupied by GFAP was evident in the EAE mice compared to the naïve animals (*p* < 0.05; Additional file 1: Fig. S2A, B, D). By contrast, no differences were found in the number of cells expressing TH in the LC (Additional file 1: Fig. S2A–C).

### The behavioral effects of rM3D(Gs)-DREADD_onset_-mediated LC activation

In order to assess the effect of chronic LC activation from the onset of the motor symptoms in EAE animals (~ 12 dpi), we evaluated the animal’s motor deficit daily from 7 dpi based on their clinical score. A significant effect on the clinical score was observed through a reduction in the AUC and a lower maximum score in the EAE-rM3D_onset_ animals relative to the EAE mice (*p* < 0.01 and *p* < 0.05, respectively; Fig. 1B, C). Disease severity was expressed as the relative change in the clinical score, which was lower in EAE-rM3D_onset_ compared with control EAE animals in the chronic phase (*p* < 0.01) and with EAE-rM3D_onset_ in the peak phase (*p* < 0.05; Fig. 1D). EAE animals suffered a significant loss in body weight along the disease course, but this was counteracted by LC activation in the EAE-rM3D_onset_ mice (*p* < 0.05; Fig. 1E).

Given that motor dysfunction affects locomotor activity in EAE animals, we tracked their spontaneous locomotor activity daily in an open field from 7 dpi, assessing the total distance travelled. As expected, EAE groups reduced their locomotor activity throughout the follow-up period relative to the naïve animals, with a decrease in the AUC (*p* < 0.001; Fig. 1F). However, chronic LC activation significantly improved the locomotor activity of EAE mice (AUC, *p* < 0.05; Fig. 1F). A subsequent analysis of the time spent in the center or the periphery of the open field highlighted the significant reduction in the time spent in the former at the peak (17 dpi) and in the chronic (25 dpi) phase of the disease (both *p* < 0.01; Fig. 1G). Interestingly, EAE-rM3D_onset_ mice spent significantly more time in the center of the field in the chronic phase (25 dpi: *p* < 0.05; Fig. 1G). The activity/attention score was also assessed as a parameter reflecting the rodent’s sickness*.* The EAE mice spent more time in a sedentary posture relative to the naïve animals at the onset (min 0–1 and 1–2, *p* < 0.05; score: *p* > 0.05; Fig. 1H) and more robustly at the peak and in the chronic phase of EAE (score: 17 dpi and 25 dpi: *p* < 0.001; Fig. 1I, J). However, EAE-rM3D_onset_ animals spent less time in this sedentary posture relative to the EAE mice, exhibiting a higher score at both the peak and in the chronic phase (score: 17 dpi: *p* < 0.05 and 25 dpi: *p* < 0.01; Fig. 1H–J).

### The effects of rM3D(Gs)-DREADD_onset_-mediated LC activation in the spinal cord

**Fig. 2:**
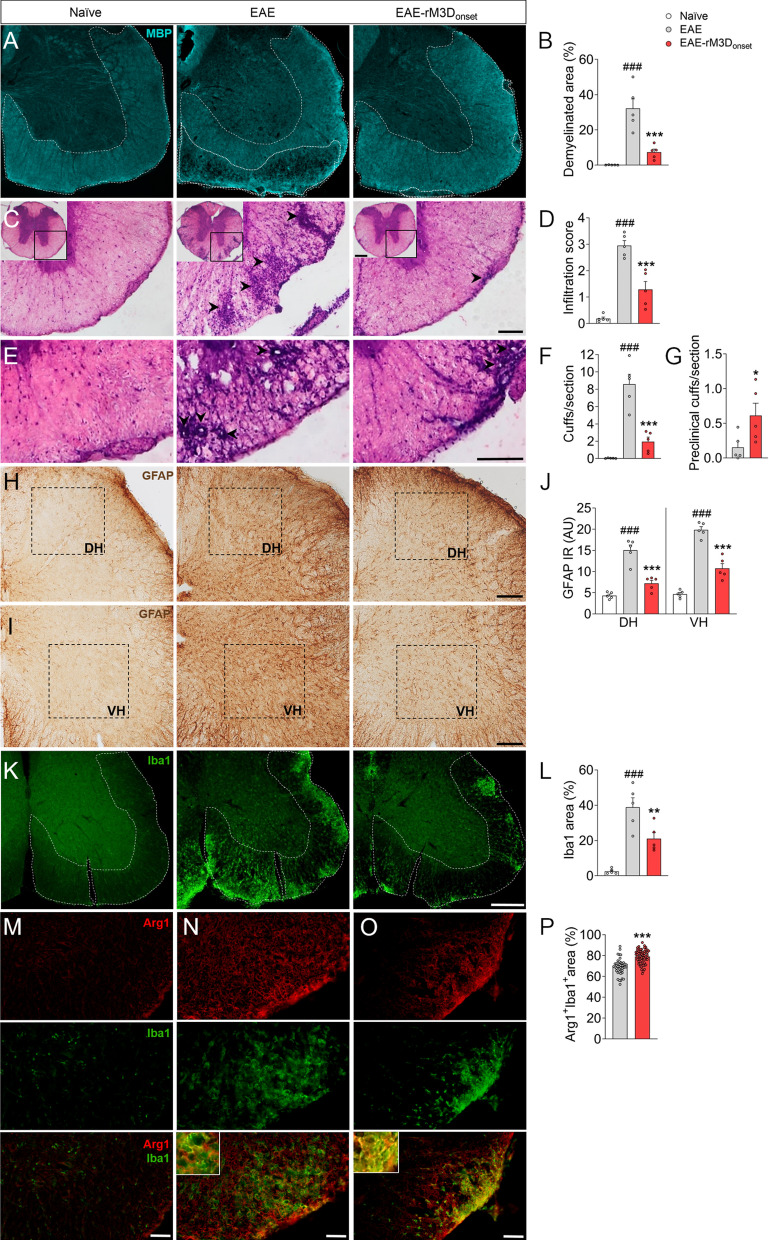
Chronic LC activation from the onset of motor symptoms affects EAE-associated damage in spinal cord. **A** Representative images showing the demyelination in the lumbar spinal cord (cyan, MBP) and **B** its quantification, expressed as the percentage of area free of MBP in the ventral white matter. **C**–**G** Perivascular infiltration in the thoracic spinal cord analyzed from **C**,** E** hematoxylin and eosin staining images and reflected as the **D** infiltration score, **F** perivascular cuffs and **G** preclinical cuffs. **H**–**J** Representative images showing astrocyte activation in the **H** dorsal and **I** ventral horns (DH, VH) of the lumbar spinal cord and a graph of the **J** GFAP immunoreactivity (arbitrary units, AU). **K** Representative immunofluorescence images showing Iba1 (green) expression in the lumbar spinal cord and **L** its quantification, expressed as the relative area of the ventral white matter expressing Iba1. **M–O** Representative immunofluorescence images of Arg1 (red) and Iba1 (green) and corresponding magnifications in the lumbar spinal cord and **P** their quantification, expressed as the percentage of area of the ventral white matter in which Arg1 and Iba1 co-localize relative to the total area occupied by Iba1. The data represent the mean + SEM and each point corresponds to an individual mouse except for **P** that represents a section (*n* = 5 per group except for **P**, 15 sections/animal (*n* = 3–4)). ^###^*p* < 0.001 EAE versus naïve; *p < 0.05, **p < 0.01, ***p < 0.001 EAE-rM3D_onset_ versus EAE (Additional file 1: Table S3). Scale bars: **A**, **K**, 200 µm; **C**,** E**,** H**,** I**, 100 µm; (**M**, **N**, **O**), 50 µm. 250 µm magnification for the insets in **C**

MBP immunoreactivity in the ventral spinal cord white matter was analyzed in the chronic phase of the disease to explore demyelination (Fig. 2A, B). In EAE mice there was a broader area of demyelination than in naïve animals (*p* < 0.001; Fig. 2B), but this was reduced significantly in EAE-rM3D_onset_ animals (*p* < 0.001; Fig. 2B). Peripheral cell infiltration was also explored and as expected, clusters of infiltrates were found in the thoracic spinal cord parenchyma of EAE mice, next to venules (*p* < 0.001; Fig. 2C, D). However, chronic activation of LC noradrenergic neurons in the EAE-rM3D_onset_ mice reduced the penetration of these infiltrates along the parenchyma of the thoracic spinal cord relative to the EAE mice (*p* < 0.001; Fig. 2D). Perivascular cuffs were also studied to determine if the infiltrates penetrate the parenchyma (Fig. 2E) and many perivascular cuffs were evident in EAE animals when compared to the naïve mice (*p* < 0.001; Fig. 2F). Fewer perivascular cuffs were found in EAE-rM3D_onset_ mice (*p* < 0.001; Fig. 2F) and more of them failed to penetrate the parenchyma (preclinical cuffs) than in EAE animals (*p* < 0.05; Fig. 2G).

Glial activation was also explored in the dorsal horn (DH) and ventral horn (VH) of the lumbar spinal cord (Fig. 2H–L), and the stronger GFAP immunoreactivity in the EAE animals’ lumbar spinal cord relative to the naïve animals (DH: *p* < 0.001; VH: *p* < 0.001; Fig. 2J) was significantly dampened in the EAE-rM3D_onset_ mice (DH: *p* < 0.001; VH: *p* < 0.001; Fig. 2J). Furthermore, there was an increase in the relative area occupied by Iba1 in EAE mice relative to naïve animals (*p* < 0.001; Fig. 2K, L), which was reduced by LC activation in the EAE-rM3D_onset_ animals (*p* < 0.01; Fig. 2L). The expression of the pro-inflammatory iNOS or the predominantly anti-inflammatory marker Arg1 were evaluated in the regions expressing Iba1. On the one hand, no iNOS immunoreactivity was found in naïve animals, yet residual iNOS expression in perivascular infiltration areas was found in both EAE and EAE-rM3D_onset_ animals but not co-localized with Iba1 (Additional file 1: Fig. S3A–C). Conversely, significant Arg1 expression was evident in the lumbar spinal cord white matter (Fig. 2M–O), which highly co-localized with Iba1 in EAE-rM3D_onset_ mice than in EAE animals (*p* < 0.001; Fig. 2P).

### The effects of rM3D(Gs)-DREADD_onset_-mediated LC activation in the cerebral cortex

**Fig. 3:**
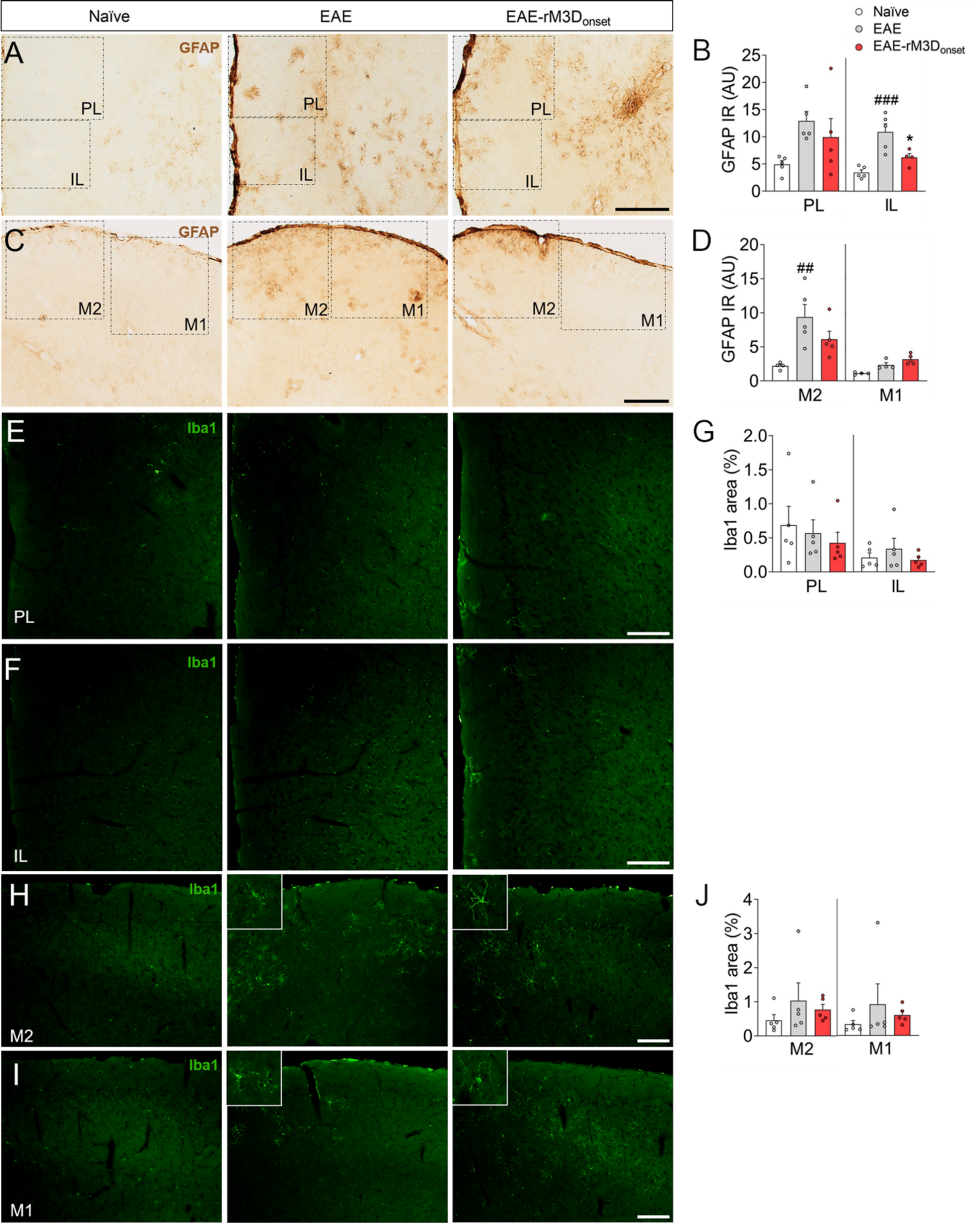
Chronic LC activation from the onset of motor symptoms affects the cortical EAE-associated glial response. Representative images showing astrocyte activation in the **A** PL/IL and **C** M2/M1 cortices, and **B**, **D** the quantification of GFAP immunoreactivity (IR) in arbitrary units (AU). **E**-**J** Microglial activation reflected by Iba1 immunofluorescence (green) in the **E** PL, **F** IL, **H** M2 and **I** M1 cortices, and **G**,** J** the relative area of these brain regions stained for Iba1. The data represent the mean + SEM and each point corresponds to an individual mouse (*n* = 4–5 per group). ^##^*p* < 0.01, ^###^*p* < 0.001 EAE versus naïve (Additional file 1: Table S4). Scale bars: **A**, **C**, 200 µm; **E**,** F**, **H**, **I**, 100 µm

Demyelination was also explored in the PFC and motor cortices, yet no differences were found in MBP expression in these areas in the chronic phase of EAE (Additional file 1: Fig. S4A-F). In terms of glial activation (Fig. 3A–D), increased GFAP expression was evident in the IL of EAE animals relative to naïve mice (*p* < 0.001; Fig. 3B) and this was reduced significantly in EAE-rM3D_onset_ mice (IL: *p* < 0.05; Fig. 3B). In the motor cortex, increased GFAP expression was evident in the M2 of EAE mice relative to the naïve animals (*p* < 0.01), but no differences were found when compared to the EAE-rM3D_onset_ mice (Fig. 3D). Regarding Iba1 expression (Fig. 3E–J), no significant differences were observed in the area of the PFC or motor cortices occupied by Iba1 in EAE animals relative to the naïve or EAE-rM3D_onset_ groups (Fig. 3G, J). Although neuroinflammation is not widespread in these cortices, we found basal activation of microglia (Iba1^+^ cells) in these brain areas but no amoeboid or macrophage-like cells were observed.

### The effect of rM3D(Gs)-DREADD_onset_-mediated LC activation on noradrenergic projections

**Fig. 4:**
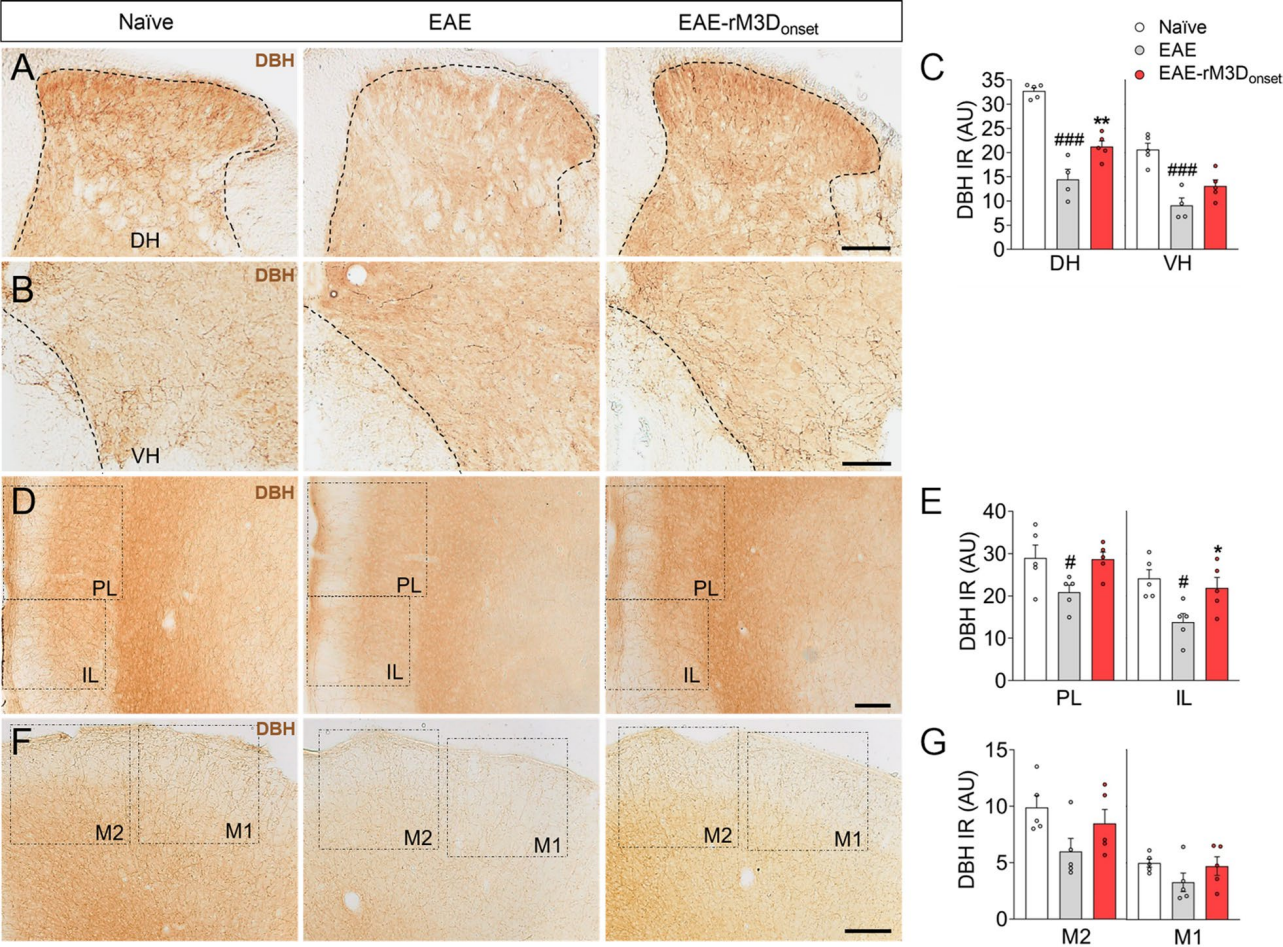
The effect of early chronic chemogenetic LC activation on noradrenergic projections. Representative images showing DBH-positive fibers in the **A** dorsal and **B** ventral horns (DH, VH) of the lumbar spinal cord, **D** PL/IL and **F** M2/M1 cortices, and **C**,** E**,** G** the quantification of DBH immunoreactivity (IR) in arbitrary units (AU). The data represent the mean + SEM and each point corresponds to an individual mouse (*n* = 4–5 per group). #p < 0.05, ###p < 0.001 EAE versus naïve; *p < 0.05, **p < 0.01, EAE-rM3D_onset_ versus EAE (Additional file 1: Table S5). Scale bars: **A**,** B**,** D**,** F**, 100 µm

DBH expression in the lumbar spinal cord was reduced all along the spinal cord gray matter in EAE animals (Fig. 4A, B) and in particular, there was a significant reduction in DBH in both the DH and VH of EAE animals relative to the naïve mice (DH: *p* < 0.001; VH: *p* < 0.001; Fig. 4C), which was reversed in the lumbar spinal cord DH by LC activation (*p* < 0.01; Fig. 4C). Like the spinal cord, there was weaker DBH expression in the PL and IL of EAE mice relative to the naïve animals (*p* < 0.05), which was significantly reversed in the IL of EAE-rM3D_onset_ mice (*p* < 0.05; Fig. 4E). Notably, no significant reduction in DBH expression was evident in the motor cortex of the EAE animals (Fig. 4G). The changes in DBH expression correlated well with GFAP immunoreactivity (Additional file 1: Fig. S5A–F) and there appeared to be a significant negative correlation between these markers in the DH and VH of the spinal cord (both *p* < 0.01), and in the IL (*p* < 0.05).

### The behavioral effects of rM3D(Gs)-DREADD_peak_-mediated LC activation

**Fig. 5:**
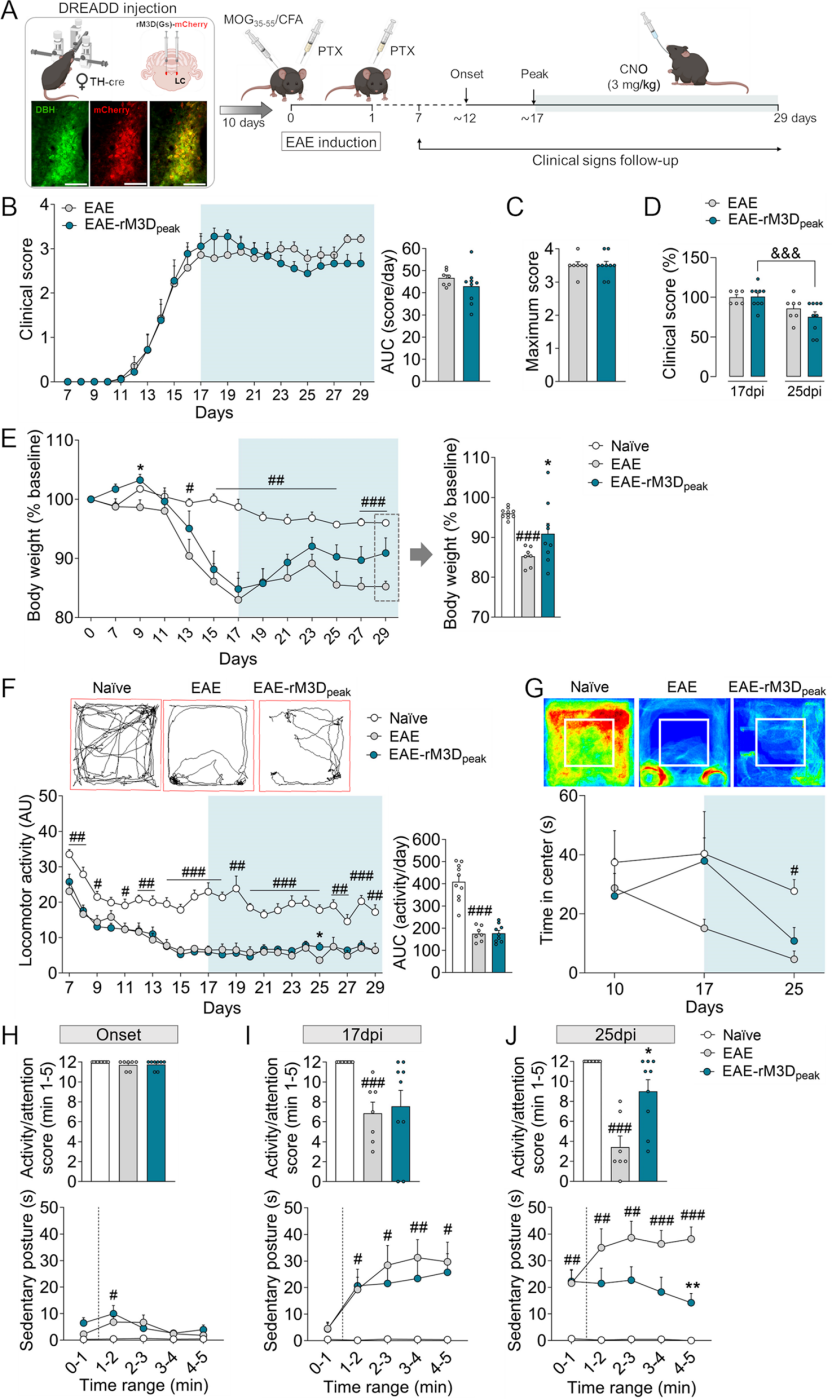
Chronic LC activation from the peak of motor symptoms affects EAE-induced behavioral alterations. **A** Experimental timeline showing the design of the study to assess the effect of chronic LC activation from the peak of motor symptoms in EAE. Representative images showing mCherry (red) expression in DBH (green) in the LC. Scale bars: 100 µm. **B** Clinical score recorded daily through the course of EAE and their area under the curve (AUC) from the peak of motor deficit and CNO administration (shaded area). **C** The severity of EAE is expressed as the maximum clinical score achieved and **D** percentage change relative to the 17 dpi EAE group, for both the peak (17 dpi) and the chronic (25 dpi) phases of EAE. **E** Body weight over the course of EAE expressed as the relative change from baseline (at the beginning of experiments) and at the end of experiments (29 dpi). **F** The representative activity traces (chronic phase) and the spontaneous locomotor activity expressed as the total distance travelled (arbitrary units, AU), and the AUC from the peak of the motor deficit and CNO administration (shaded area). **G** Representative heat maps (chronic phase) and the relative time spent in the central area of the arena before the onset of motor deficit and CNO administration (10 dpi), at the peak (17 dpi) and chronic (25 dpi) phases of EAE. **H**–**J** Activity/attention score (above) and the time spent in the sedentary posture (below) at **H** the onset, **I** the peak (17 dpi) and **J** in the chronic (25 dpi) phase of EAE. The data represent the mean + SEM and each point corresponds to an individual mouse (*n* = 6–10 per group). ^#^*p* < 0.05, ^##^*p* < 0.01, ^###^*p* < 0.001 EAE versus naïve; *p < 0.05, **p < 0.01 EAE-rM3D_onset_ versus EAE; ^&&&^*p* < 0.001 EAE-rM3D_onset_ 25 dpi versus 17 dpi (Additional file 1: Table S6). Some elements of this figure were created with BioRender.com

The effect of chemogenetic LC activation was explored from the peak of motor symptoms (~ 17 dpi), when animals exhibited robust hind limb paralysis (Fig. 5A). In terms of the clinical signs, no significant differences were found in the motor symptomatology of the EAE-rM3D_peak_ mice relative to the EAE animals during the development of the disease, or even in the AUC and the maximum score attributed to these animals (Fig. 5B, C). However, the clinical score of EAE-rM3D_peak_ animals was significantly lower in the chronic phase (25 dpi, *p* < 0.001; Fig. 5D), indicating an improvement to the motor deficits after approximately 8–10 days of LC activation. In addition, LC activation significantly restored the loss in body weight induced by EAE in the chronic phase (*p* < 0.05, EAE-rM3D_peak_ vs EAE; Fig. 5E).

When the effect of LC activation on spontaneous locomotor activity was explored, there was a significant improvement at 25 dpi (*p* < 0.05), although the AUC score was similar between EAE and EAE-rM3D_peak_ mice (Fig. 5F). Furthermore, no significant differences were found in anxiety-like behavior in the EAE-rM3D_peak_ animals, measured as the time spent in the central area of the open field (Fig. 5G). In terms of the activity/attention score (Fig. 5H–J), EAE-rM3D_peak_ animals recover the activity/attention score in the chronic phase because they spent less time in this sedentary posture (25 dpi: score: *p* < 0.05; min 4–5, *p* < 0.01; Fig. 5J).

### The effects of rM3D(Gs)-DREADD_peak_-mediated LC activation in the spinal cord

**Fig. 6:**
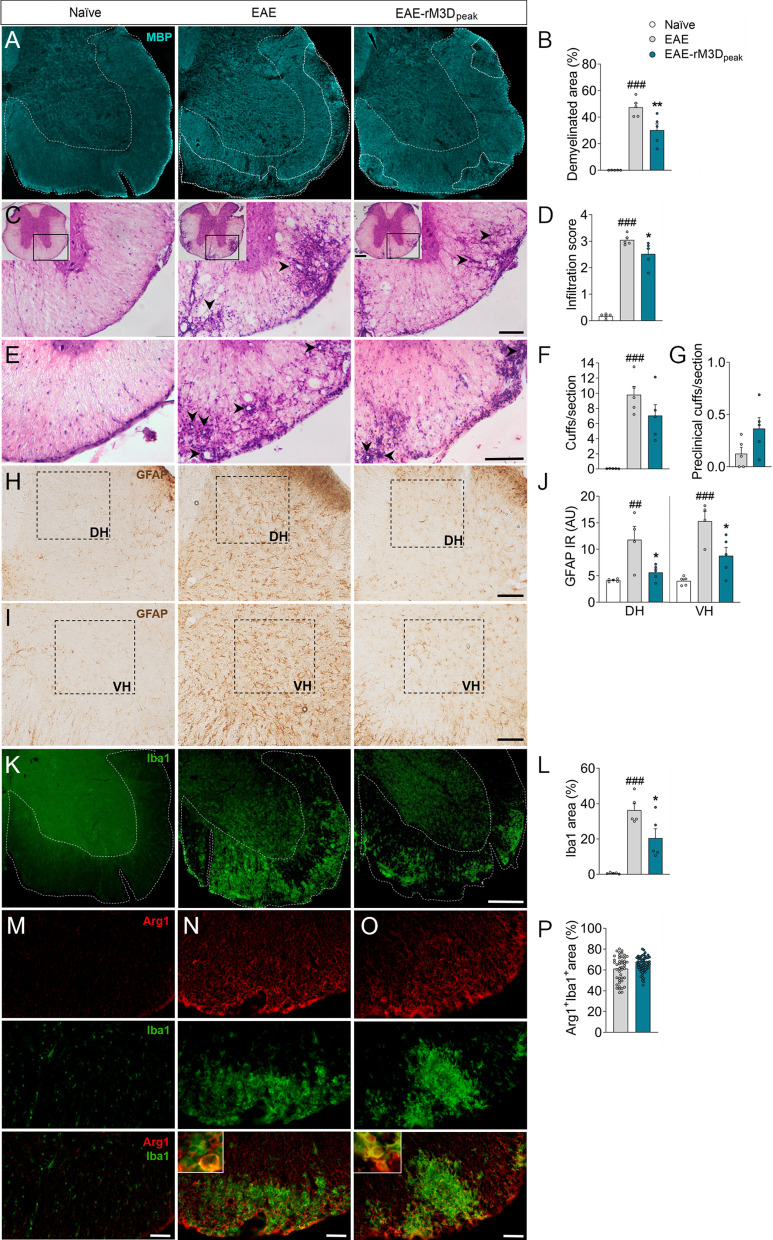
Chronic LC activation from the peak of the EAE motor symptoms affects the spinal cord. **A** Representative images showing the demyelination in the lumbar spinal cord (cyan, MBP) and **B** its quantification expressed as the percentage of area free of MBP in the ventral white matter. **C**–**G** Perivascular infiltration in the thoracic spinal cord analyzed from **C**, **E** hematoxylin and eosin staining images and reflected as the **D** infiltration score, **F** perivascular cuffs and **G** preclinical cuffs. **H**–**J** Representative images showing astrocyte activation in the **H** dorsal and **I** ventral horns (DH, VH) of the lumbar spinal cord, and a graph of the **J** GFAP immunoreactivity (IR) in arbitrary units (AU). **K** Representative immunofluorescence images showing Iba1 (green) expression in the lumbar spinal cord and **L** its quantification, expressed as the relative area of the ventral white matter expressing Iba1. **M–O** Representative immunofluorescence images of Arg1 (red) and Iba1 (green) and corresponding magnifications in the lumbar spinal cord and **P** their quantification, expressed as the percentage of area of the ventral white matter in which Arg1 and Iba1 co-localize relative to the total area occupied by Iba1. The data represent the mean + SEM and each point corresponds to an individual mouse for **P** that represents a section (*n* = 4–5 per group except for **P**, 15 sections/animal (*n* = 3–4)). ^##^*p* < 0.01; ^###^*p* < 0.001 EAE versus naïve; *p < 0.05, **p < 0.01 EAE-rM3D_peak_ versus EAE (Additional file 1: Table S7). Scale bars: **A**, **K**, 200 µm; **C**,** E**,** H**, **I**, 100 µm; **M**, **N**, **O**, 50 µm. 250 µm insets in **C**

Despite the fact that CNO administration commenced when demyelination was quite advanced (Fig. 6A, B), there was a smaller demyelinated area in the ventral white matter of the lumbar spinal cord in EAE-rM3D_peak_ mice than in the EAE animals (*p* < 0.01; Fig. 6B). Furthermore, there was less penetration of perivascular infiltrates along the parenchyma in the thoracic spinal cord of EAE-rM3D_peak_ mice than in EAE animals (*p* < 0.05; Fig. 6C, D). Nevertheless, there were no differences in the number of perivascular cuffs (Fig. 6E, F) or in the number of preclinical perivascular cuffs (Fig. 6G) between EAE-rM3D_peak_ and EAE animals.

As in EAE-rM3D_onset_ mice, GFAP immunoreactivity was weaker in the DH and VH of the lumbar spinal cord in EAE-rM3D_peak_ mice relative to the EAE animals (DH: *p* < 0.05; VH: *p* < 0.05; Fig. 6H–J). Similarly, the relative area occupied by Iba1 in the ventral white matter of the lumbar spinal cord was smaller in EAE-rM3D_peak_ than in the EAE animals (*p* < 0.05; Fig. 6K, L). Finally, there was residual iNOS expression in both EAE and EAE-rM3D_peak_ mice, yet this did not co-localize with Iba1 (Additional file 1: Fig. S3D-F). Furthermore, there were no differences in Arg1 and Iba1 co-localization in EAE-rM3D_peak_ and EAE mice (Fig. 6M–P).

### The effects of rM3D(Gs)-DREADD_peak_-mediated LC activation in the cerebral cortex

**Fig. 7:**
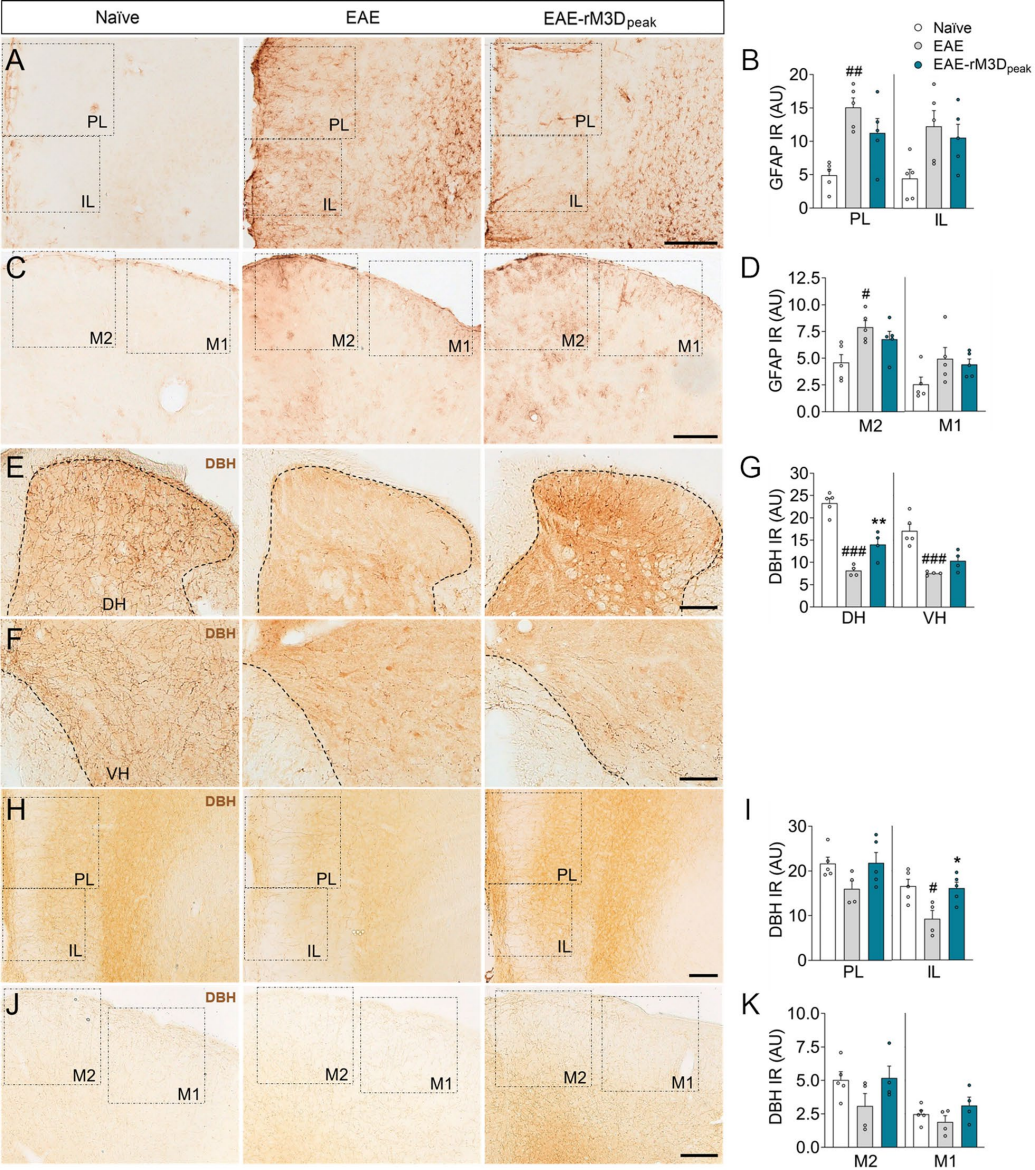
The effect of late chronic LC activation on cortical astrocyte activation and noradrenergic projections. Representative images showing astrocyte activation in the **A** PL/IL and **C** M2/M1 cortices, and **B**, **D** the quantification of GFAP immunoreactivity (IR) in arbitrary units (AU). Representative images showing DBH positive fibers in **E** the dorsal and **F** ventral horns (DH, VH) of the lumbar spinal cord, **H** PL/IL and **J** M2/M1 cortices, and **G**, **I**,** K** the quantification of DBH IR in AU. The data represent the mean + SEM and each point corresponds to an individual mouse (*n* = 4–5 per group). ^#^*p* < 0.05; ^###^*p* < 0.001 EAE versus naïve; *p < 0.05, **p < 0.01 EAE-rM3D_peak_ versus EAE (Additional file 1: Table S8). Scale bars: **A**, **C**, 200 µm; **E**,** F**,** H**,** J**, 100 µm

We did not observe any significant differences in GFAP expression in the PFC between EAE-rM3D_peak_ and EAE mice (Fig. 7A, B), or in the motor cortex (Fig. 7C, D).

### The effect of rM3D(Gs)-DREADD_peak_-mediated LC activation on noradrenergic projections

Stronger DBH expression was evident in the DH of the spinal cord in EAE-rM3D_peak_ mice relative to the EAE animals (*p* < 0.01; Fig. 7E–G), and in the IL (*p* < 0.05; Fig. 7H, I). However, no significant differences were evident in the M2/M1 cortex of EAE mice relative to the naïve or EAE-rM3D_peak_ animals (Fig. 7J, K). Moreover, a correlation analysis between DBH and GFAP immunoreactivity showed a significant negative correlation in the DH (*p* < 0.05) and VH of the spinal cord (*p* < 0.01; Additional file 1: Fig. S5G–L).

## Discussion

The EAE model recapitulates some key aspects of MS and in agreement with previous findings, it provokes time dependent motor weakness and paralysis, as well as other non-motor symptoms. In the CNS, demyelination, inflammation and glial activation in the spinal cord and to a lesser extent in the PFC and motor cortices, was evident in the chronic phase of EAE [37, 38], in conjunction with reduced DBH expression in these areas. Furthermore, enhanced GFAP expression was found in the LC of EAE animals, although the number of TH expressing cells does not change. As previous data demonstrated anti-inflammatory and neuroprotective effects of noradrenaline [15], the enhanced CNS noradrenergic tone after activation of the LC was explored. When chemogenetic LC activation was induced at the onset of motor symptoms, the animal’s symptoms improved greatly, such as their anxiety and activity/attention score, as well as their motor deficits. This was accompanied by a clear reversion of demyelination and glial activation in the spinal cord, along with a dampening of the inflammatory parameters, and of DBH expression in the spinal cord and PFC. A second chemogenetic approach involved LC activation when the animals were experiencing more severe paralysis, which demonstrated benefits to animal well-being and a modest alleviation of motor symptoms, as well as an improvement in demyelination and glial activation in the spinal cord, along with an increase in DBH expression in the spinal cord and PFC.

When the possible alterations to the LC induced by EAE were assessed, there was a clear increase in the area of GFAP expression in the LC of EAE animals, which could be related to the role of astrocytes in the neural plasticity that may be induced in the LC projecting areas. However, the number of TH expressing cells remained unaltered at this chronic phase of EAE, consistent with previous findings pointing to an absence of neuronal loss in the LC [9]. When the effect of chemogenetic LC activation was explored from the onset of motor symptoms (~ 12 dpi), the severity of motor dysfunction induced by EAE was alleviated, as witnessed through the clinical score. Spontaneous locomotion was also evaluated in the open field test in which animals can move freely in an arena for 5 min. As expected, the locomotor activity of EAE animals was worse than that of naïve animals as EAE progressed, although it increased significantly in EAE-rM3D_onset_ mice. Interestingly, EAE animals prefer to move to the periphery, avoiding the central anxiogenic area, whereas the EAE-rM3D_onset_ mice better explored the central area, especially after long-term CNO treatment. This is consistent with previous data showing that non-motor symptoms of MS arise, like anxiety [39, 40], and now we show that activation of the LC can help to relieve these alterations. Indeed, this improvement is in agreement with the anxiolytic effect of chronic treatment with noradrenaline enhancing drugs [41, 42]. The activity/attention score was considered a parameter of sickness in rodents and it is significantly reduced in EAE mice [34]. This score is related to the time spent in a specific sedentary posture, reflecting a loss of interest for the environment. This parameter improved considerably in EAE-rM3D_onset_ mice, which spent less time in this sedentary posture both in the peak and chronic phases of EAE. These data indicate that chronic LC activation has a clear beneficial effect on the non-motor symptoms induced by EAE. In addition, LC activation reduced the weight loss induced by EAE, another sign of animal welfare.

One of the most important characteristic of the EAE model is the acute demyelination that contributes to the motor impairment suffered by these mice, which was clearly dampened by rM3D(Gs)-DREADD_onset_**-**mediated LC activation**.** Demyelination is related to the permeabilization of the blood–brain barrier which allows cells from the peripheral immune system to access to the CNS parenchyma [43] as witnessed by the peripheral infiltrates in the spinal cord parenchyma next to venules in EAE animals. However, we found that chronic LC noradrenergic neurons activation reduced the clusters of infiltrates along the parenchyma in the EAE-rM3D_onset_ spinal cord relative to that in EAE mice. The penetration of these infiltrates through the endothelium into the parenchyma is thought to occur in two phases. First, infiltrates pass through the laminin-containing endothelial basement membrane, the membrane proximal to the endothelium. Some days later they penetrate the parenchymal basement membrane, abutting the CNS parenchyma [44, 45] but while the infiltrates gather in the space between these two basement membranes, known as the perivascular space, they form cuffs. These perivascular cuffs can be considered to be clinical if the infiltrates have penetrated the parenchyma or preclinical if they have not yet penetrated the parenchyma. LC activation reduced perivascular infiltration in the spinal cord parenchyma with a predominance in the presence of preclinical cuffs compared to EAE. Hence, activation of the noradrenergic LC when motor symptoms commence leads to a clear reduction in EAE behavioral symptomatology, while also dampening its pathophysiological progression in terms of spinal cord demyelination and perivascular infiltration.

While glial and immune cells take part in the neuroinflammatory events associated with EAE, either exacerbating or driving their resolution, LC activation dampened the increase in GFAP immunoreactivity in the EAE animal’s spinal cord. The area occupied by Iba1 reflects the activated microglia and infiltrated macrophages that tend to migrate to damage zones to remove cellular debris, such as that produced by demyelination [46]. The increase in the relative area occupied by Iba1 in the ventral white matter of the spinal cord in EAE animals was dampened by the LC activation in EAE-rM3D_onset_ animals. This microglial activation was assessed in the relation to the pro-inflammatory marker iNOS or the predominantly anti-inflammatory marker Arg1. Residual iNOS expression was evident in both EAE and EAE-rM3D_onset_ animals but it did not co-localize with Iba1 which suggests that this inflammatory mechanism is not present at this temporal point. Nevertheless, inflammation is thought to fulfill a critical regulatory role in the early stages of EAE, both during the antigen-priming phase and in the subsequent effector phase of the immune response in which the spinal cord is mainly involved [47]. Alternatively, we found enhanced Arg1 expression in spinal cord white matter and stronger co-localization between Arg1 and Iba1 in the EAE-rM3D_onset_ animals than in EAE mice. Thus, LC activation from the onset of motor symptoms may promote the expression of anti-inflammatory markers that contribute to neuroprotection and/or to the resolution of inflammatory events like reactive gliosis. To correlate these behavioral and pathogenic observations with the LC-noradrenergic system, DBH expression was examined in the spinal cord. EAE animals have weaker DBH expression in this tissue and this was echoed by a diminished density of DBH immunoreactive fibers. However, the activation of LC noradrenergic neurons reversed the DBH deficit in the spinal cord (mainly in the DH). Interestingly, a significant negative correlation was found between DBH and GFAP expression in the spinal cord and IL. Hence, the astroglial reaction induced by EAE may be associated with a reduction in DBH expression, such that LC activation was able to counteract this imbalance, suggesting an inverse relationship between glial activation and the lower density of DBH immunoreactive fibers in these areas. This might explain why some therapeutic approaches using noradrenergic precursors are not always effective, such as the use of L-phenylalanine that participates in the DBH pathway [27, 28], while others do produce positive effects in EAE mice, for example the administration of L-DOPS that is decarboxylated to noradrenaline by endogenous L-aromatic-amino-acid decarboxylase [10]. Therefore, noradrenergic strategies that use alternative DBH routes or that promote DBH activity (such as LC chemogenetics) may be more efficient therapeutic approaches.

In addition to the spinal cord, the PFC (PL and IL) and motor cortices of EAE animals were explored since there is evidence of noradrenaline deficits at cortical levels [9]. Changes in neurodegeneration and glial markers were substantially less severe than in the spinal cord suggesting that these brain areas are better preserved. However, there was a clear reduction in DBH expression in the PL and IL of EAE animals in the chronic phase that seems to be prevented by LC activation in the IL, and more modestly (but not significantly) in the PL. These data are relevant as deficient noradrenergic innervation has been related to anxiodepressive disorders [48], and this may therefore be related to the improvements in sickness behavior and anxiety.

The data obtained agree overall with the previous observations regarding the anti-inflammatory and neuroprotective actions of noradrenaline. Noradrenaline exerts an anti-inflammatory effect through glial cells by attenuating the expression of pro-inflammatory mediators in response to inflammatory events (e.g., TNF-α, IL-1β, NO, iNOS or NFκB) [17, 49–53]. Noradrenaline enhancement also promotes neurotrophic support and myelin production through the activation of β1/2- and α1-adrenoceptors in astrocytes and oligodendrocytes, respectively [54–56] and it reduces CNS chemokines and cell adhesion molecules expression, molecules that facilitate leucocyte influx into the CNS [57]. These mechanisms triggered by noradrenaline would explain the dampened neuroinflammation and perivascular infiltration observed here after LC noradrenergic activation, as well as the alleviation or prevention of the EAE associated demyelination. Furthermore, modulation of the immune system might be underlying the beneficial effects of noradrenergic tone augmentation. Hence, adrenergic signaling usually acts as a potent suppressive pathway on innate immune cells such as macrophages and dendritic cells [58, 59], mainly through β2-adrenoceptors, suppressing pro-inflammatory cytokines secretion. Moreover, noradrenaline suppresses toll-like receptor pathways blocking NFκB activation and increases IL-10 expression that carries out autocrine signaling to block TNF-α and other pro-inflammatory cytokines expression [58–62]. Thus, IL-10 may play a key role in the anti-inflammatory properties of noradrenaline suppressing local and systemic inflammatory processes [62]. On the other hand, noradrenaline and β-agonists are able to regulate T cell effector functions, for example, suppressing CD8^+^ subsets expression of IFN-γ and TNF-α [63]. Therefore, noradrenaline seems to exert anti-inflammatory effect on immune cells predominantly by its interaction with β-adrenergic receptors.

Once the beneficial effect of chronic LC activation from the onset of motor symptoms was demonstrated in EAE animals, a more challenging approach was adopted to test LC activation from the peak of motor symptoms (~ 17 dpi), when animals exhibited a robust hind limb paralysis. LC activation for 12–15 days from the peak of motor symptoms only led to a slight alleviation of the motor symptomatology induced by EAE, and although no effect was seen on anxiety, a clear improvement in sickness behavior was evident, as well as an alleviation of body weight loss in the chronic phase of EAE. When exploring demyelination, LC activation significantly reduced the relative size of the demyelinated area in the ventral white matter of the spinal cord, in conjunction with less penetration of perivascular infiltrates along the parenchyma. Nevertheless, no differences in the number of perivascular cuffs or in the number of preclinical perivascular cuffs were found in EAE-rM3D_peak_ mice relative to the EAE animals. As the treatment started at the peak of motor symptoms, infiltration was very advanced and the CNS parenchyma was probably already abutted. However, the treatment seemed to prevent further dissemination of these infiltrates along the CNS parenchyma. Likewise, there was weaker GFAP immunoreactivity in the spinal cord of EAE-rM3D_peak_ animals, which correlates with an increase in DBH expression, indicating that beneficial effects induced by LC chemogenetic activation is possible even at advanced stages of EAE progression. Iba1 immunoreactivity in the spinal cord was also weaker in the EAE-rM3D_peak_ mice but it did not co-localize with iNOS. Moreover, the co-expression of Iba1 with Arg1 was similar to that in EAE animals, probably due to LC activation beginning at a later stage of EAE, when inflammation is already advanced. In addition, this chemogenetic LC activation failed to impede cortical EAE-induced GFAP expression even though stronger DBH expression was found in the IL cortex.

## Conclusions

These two chemogenetic approaches demonstrated that the noradrenergic LC may be selectively activated even at advanced stages in the evolution of the disease, which suggests that the LC cellular machinery is not so severely affected as in other neurodegenerative pathologies [25]. Although beneficial effects have been achieved through experimental approaches to enhance noradrenaline availability in both preclinical and clinical studies [10, 27, 28, 30, 34, 64–68], we demonstrate here that the selective activation of LC noradrenergic neurons can alleviate EAE symptomatology. When chemogenetic LC activation starts early, there is a clear neuroprotective and anti-inflammatory effect in the spinal cord, one of the critical areas for the MS, as well as in the IL, which correlates with the benefits to the motor and non-motor symptoms of the disease. The second and more challenging approach arises when the chemogenetic LC activation commences once hind limb paralysis is evident, again producing benefits at the motor and non-motor level that are specifically associated with weaker pathological changes at the spinal cord and IL. It is relevant to note that this treatment is shorter, such that delayed beneficial effects might become evident at later time points. Overall the data suggest that the beneficial effects of the chemogenetic activation of the LC in the context of EAE reflect a potent anti-inflammatory and neuroprotective response. The decrease in reactive gliosis, as well as infiltrated cells and potentiation of the anti-inflammatory response in Iba1^+^ cells, leads to the interpretation of a synergistic signal between the different cell types that converge in a reduction of the pro-inflammatory response and potentiation of the anti-inflammatory one, which facilitates the resolution of the inflammation [15, 69]. Therefore, further studies are necessary to discern how chronic LC activation modulates the pro- or anti-inflammatory profile in EAE along to the identification of the immune populations participating in the therapeutic effect (Th1, Th2, Th17, CD11b, CD45, CD68, CD80, CD163, CD206, MCP1 lymphocytes) that might become key pieces of MS and its clinical management. Finally, this study shows that there is a substantial window of opportunity for LC/noradrenaline-based therapies, even at advance phases of the disease.

### Supplementary Information

**Additional file 1. **This file includes a detailed description of materials and methods, supplementary figures (Figs. S1–S5) and supplementary tables for antibodies used in this study (Table S1) and summarizing the statistical analysis (Tables S2–S11).

## Data Availability

All datasets used and/or analyzed during the current study are available within the article and its Supplementary Information. Extra information about the data is available on request to the corresponding author.

## Abbreviations

ANOVA: Analysis of variance
AP: Anteroposterior
AU: Arbitrary units
AUC: Area under the curve
Arg1: Arginase-1
CFA: Complete Freund’s adjuvant
CNO: Clozapine-N-oxide
CNS: Central nervous system
DBH: Dopamine beta-hydroxylase
DH: Dorsal horn
dpi: Days post EAE induction
DREADD: Designer receptors exclusively activated by designer drugs
EAE: Experimental autoimmune encephalomyelitis
GFAP: Glial fibrillary acidic protein
Iba1: Ionized calcium-binding adapter molecule 1
iNOS: Inducible nitric oxide synthase
IL: Infralimbic cortex
LC: Locus coeruleus
L-DOPS: L-threo-3,4-Dihydroxyphenylserine
M1: Primary motor cortex
M2: Secondary motor cortex
MBP: Myelin basic protein
ML: Midline
MOG_35–55_: Myelin oligodendrocyte glycoprotein peptide 35–55
MS: Multiple sclerosis
PFC: Prefrontal cortex
PL: Prelimbic cortex
PTX: Pertussis toxin
SEM: Standard error of the mean
TH: Tyrosine hydroxylase
VH: Ventral horn
