# Supplementary material for: Chemogenetic activation of locus coeruleus neurons ameliorates the severity of multiple sclerosis
Source: J Neuroinflammation. 2023 Sep 1;20:198. doi: 10.1186/s12974-023-02865-z (PMC10474779; doi:10.1186/s12974-023-02865-z)
Supplement: Supplementary file 1 — Additional file 1. This file includes a detailed description of materials and methods, supplementary figures (Figs. S1–S5) and supplementary tables for antibodies used in this study (Table S1) and summarizing the statistical analysis (Tables S2–S11). [file 12974_2023_2865_MOESM1_ESM.pdf]

Chemogenetic activation of locus coeruleus neurons ameliorates the severity of multiple sclerosis

Alejandro Torrillas-de la Cal[1,2,3†], Sonia Torres-Sanchez[1,2,3†], Lidia Bravo[1,2,3], Meritxell Llorca-Torralba[2,3,4], Jose Antonio Garcia-Partida[1,2,3], Ana I. Arroba[3,5], Esther Berrocoso[1,2,3*].

[†]These authors have contributed equally to this work and share first authorship.

[1]Neuropsychopharmacology & Psychobiology Research Group, Department of Neuroscience, University of Cádiz, 11003 Cádiz, Spain.

[2]Ciber de Salud Mental (CIBERSAM), Instituto de Salud Carlos III, 28029 Madrid, Spain.

[3]Instituto de Investigación e Innovación Biomédica de Cádiz (INiBICA), Hospital Universitario Puerta del Mar, 11009 Cádiz, Spain.

[4]Neuropsychopharmacology & Psychobiology Research Group, Department of Cell Biology & Histology, University of Cádiz, 11003 Cádiz, Spain.

[5]Department of Biomedicine, Biotechnology and Public Health (Immunology Area), University of Cádiz, 11003 Cádiz, Spain.

***Correspondence:**
Esther Berrocoso
E-mail address: esther.berrocoso@uca.es; telephone number: +34 956015224

Additional file 1

Materials and methods

Animals

Female C57BL/6J TH:Cre mice, 10 weeks old, were maintained under standard laboratory conditions (22 ºC, 12 h light/dark cycle, lights on at 08:00 am, food and water *ad libitum*) at the University of Cadiz. The TH:Cre founders were provided by INFRAFRONTIER/EMMA (EM:00254) [1] (kindly donated by T. Ebendal, Karolinska Institutet, Stockholm, Sweden). All animal handling and the procedures were carried out in accordance with the European Commission guidelines (2010/63/EU) and Spanish Law (RD 53/2013) regulating animal research. Moreover, the protocols were approved by the Ethics Committee for Animal Experimentation at the School of Medicine of the University of Cadiz (Spain).

Experimental design and groups

The experimental designs are shown in Fig. 1A and Fig. 5A. Briefly, 10-week-old mice were administered with Designer Receptor Exclusively Activated by Designer Drugs (DREADD) virus bilaterally into the LC. After a 10 day recovery, EAE was induced and the clinical symptoms were monitored from day 7 post EAE induction (dpi) from 09:00 am to 02:00 pm. Chronic chemogenetic LC activation was achieved by orally gavage of clozapine-N-oxide (CNO, 3 mg/kg daily) from the onset of EAE (~12 dpi) or the peak of the motor symptoms (peak phase: clinical score ≥ 3, ~17 dpi). Finally, the mice were sacrificed in the chronic phase of EAE (from ~20 dpi), collecting the spinal cord and brain tissues (27-29 dpi) for neurobiological assessment. The experimental groups studied were healthy control animals (naïve group) and EAE animals previously administered control-DREADD or rM3D(Gs)-DREADD virus into LC (EAE and EAE-rM3D groups, respectively). All the EAE mice were treated chronically with CNO (from the onset or the peak of the motor symptoms) and the naïve animals received the vehicle alone (drinking water). Mice were assigned to different treatment groups in a randomized manner. The sample size was calculated using the G*Power tool, based on similar published studies. Experimenters were blind to the treatment.

DREADD virus injection

Mice were anesthetized with isoflurane (4% for induction and 2% for maintenance), placed into a stereotaxic frame and the DREADD virus was injected bilaterally (0.5 µl/side) into the LC (anteroposterior [AP] -5.3 mm, midline [ML] ±1 mm relative to bregma and -3.6 mm from the surface of the skull) [2]. The DREADD vectors used (Gene Therapy Center Vector Core, University of North Carolina, USA) were: AAV2/hSyn-DIO-mCherry (control-DREADD; titer 5.6 x 10^{12} vg/ml) and AAV2/hSyn-DIO-rM3D(Gs)-mCherry (rM3D(Gs)-DREADD, activator

virus, titer 3 x 10^{12} vg/ml). The control vector contained a mCherry reporter protein without a DREADD receptor. To enhance vector expression and favor the animals' recovery, EAE induction was carried out 10 days later. At the end of experiments, the expression of DREADD in the LC was verified by immunofluorescence (Fig. S1).

EAE induction and evaluation

After DREADD administration, mice (12-weeks-old) were immunized subcutaneously in the upper and lower back areas with an emulsion containing 200 µg of mouse myelin oligodendrocyte glycoprotein peptide ($MOG_{35–55}$) in complete Freund's adjuvant (CFA). The mice were also administered 100 ng of pertussis toxin in PBS intraperitoneally (PTX) 2 and 24 hours later (#EK-2110, Hooke Laboratories Inc, USA). Immunization was carried out under isoflurane anesthesia and the control animals were subjected to the same procedure but administered PBS alone. As soon as the first signs of paralysis appeared, the mice were provided with easily accessible water, wet food pellets and jelly with sucrose on the floor of the cage to ensure proper nutrition and hydration, even to animals with severe paralysis.

Clinical signs: All mice were monitored daily for clinical signs of EAE and every two days their body weight was controlled blind to the treatment. The mice were scored using the following scale (clinical score): 0, no clinical signs; 0.5, partially limp tail; 1, limp tail; 1.5, limp tail and imbalance; 2, limp tail and moderate hind limb weakness; 2.5, limp tail and partial paralysis in at least one hind limb; 3, limp tail and complete paralysis of both hind limbs; 3.5, limp tail, complete hind limb paralysis and weakness in the forelimbs (able to right itself when placed on its side); 4, limp tail, complete hind limb paralysis and weakness in the forelimbs (unable to right itself when placed on its side); 4.5, limp tail and paralysis in all the limbs (quadriplegia, end-point criteria). The area under the curve (AUC) values of the clinical score, the maximum score achieved and the relative change in clinical score at the peak (17 dpi) and in the chronic (25 dpi) phase of EAE (relativized to EAE group at the peak phase) were also analyzed.

Open field test: Mice were placed individually in a square arena (45 × 45 × 35 cm) that they were allowed to explore freely for 5 min. The sessions were videotaped and analyzed offline, using SMART (Spontaneous Motor Activity Recording and Tracking) video 3.0 software (Panlab, Spain). Spontaneous locomotor activity was measured as the total distance travelled, expressed in arbitrary units (AU) and through the AUC values of the total distance travelled. The time spent in the central area of the arena was also measured as a readout of anxiety-like behavior. Animals were placed in the center of the arena and those that failed to move out of the central region were not considered for the trial. Finally, the activity/attention score was evaluated over the last 4 min of free exploration period. This score was related to the "sickness" of the mice and it was assessed through the time spent in a specific sedentary posture that is characterized by the placement of

both forepaws on the ground and maintaining the head relatively still with a steady gaze directed below horizontal [3]. The activity/attention score was based on the following criteria regarding the time spent in this position in each minute of the 4 minutes test period: 0 (45-60 s), 1 (30-45 s), 2 (15-30 s) and 3 (0-15 s).

Histology, immunohistochemistry and immunofluorescence assays

Animals were perfused with paraformaldehyde (4%) at the end of the experiments, and their brain and spinal cord were collected, post-fixed (2 hours) and preserved at 4°C in sucrose (30% in 0.1 M phosphate-buffered saline, PBS). Coronal sections were obtained on a Sliding Microtome (Microm HM 450: Fisher Scientific SL, Spain) coupled to BFS-MP freezing stage for microtome (Physitemp Instruments, USA), that contained the lumbar spinal cord (50 µm, 1 in every 5 sequential sections), the prefrontal cortex (PFC): prelimbic (PL) and infralimbic (IL), motor cortices: secondary (M2) and primary (M1) and LC, A5 and A7 (40 µm, 1 in every 6 sequential sections). In addition, coronal sections obtained on a Microm HM 525 cryostat (Thermo-Scientific, Germany) and containing 1 cm of the thoracic spinal cord region (20 µm, 1 in every 4 sequential sections) were also collected on glass slides. For immunohistochemistry and immunofluorescence assays, detailed information about the primary and secondary antibodies used are included in Table S1, and the assays were quantified using Fiji Image software (USA).

Noradrenergic cells and astrocytes in the LC: Sections containing the LC, from other set of naïve and EAE animals (27-29 dpi), were incubated for 2 nights at 4ºC with a mouse antibody against glial acidic fibrillary protein (GFAP, 1:1000; Millipore, Spain) and a sheep antibody against tyrosine hydroxylase (TH, 1:1000, Abcam, Spain). The binding of the antibodies was then detected with an Alexa Fluor 568 conjugated donkey anti-mouse (1:1000; Invitrogen™, USA) and an Alexa Fluor 647 conjugated donkey anti-sheep (1:1000; Invitrogen™, USA) antibodies. After washing and mounting in fluoro-gel aqueous medium, images were acquired on a Zeiss LSM 880 Confocal microscope using FAST Airyscan (Carl Zeiss Microscopy GmbH, Germany), and the number of TH^+ cells in the LC per field and the area occupied by GFAP (%) were assessed.

Perivascular infiltration in the spinal cord: Perivascular infiltration of the CNS parenchyma can be analyzed by hematoxylin-eosin staining. Thus, sections of the thoracic spinal cord were collected on glass slides (5 animals per group) and stained with hematoxylin-eosin following standard procedures. The images were visualized on an Olympus BX60 microscope and acquired with an Olympus DP74 camera (Spain). Perivascular infiltration in spinal cord sections was scored using the following scale: 0, no infiltration; 1, a few scattered inflammatory cells; 2, perivascular infiltrates; 3, perivascular extension into the parenchyma; and 4, extensive cell infiltration in the white matter [4]. Moreover, perivascular cuffs were counted in the spinal cord

discriminating the number of those in which infiltrates did not penetrate into the parenchyma (preclinical cuffs) [5, 6].

Demyelination in the spinal cord and cortex: In order to evaluate the demyelination in the lumbar spinal cord white matter and cortex, sections (4-5 animals per group) were subjected to antigen retrieval to unmask the epitopes. Thus, prior to probing with the antibodies, the sections were treated with a 2N HCl solution at 37°C (20 min) and then left at room temperature for 5 min. The sections were then probed for 2 nights at 4ºC with a mouse antibody against myelin basic protein (MBP, 1:1000; Biolegend, USA). Antibody binding was then detected with a biotinylated donkey anti-mouse IgG (1:200; Jackson ImmunoResearch Europe, UK) followed by Alexa Fluor 568 streptavidin (1:1000; Invitrogen^{TM}, USA). After washing and mounting in fluoro-gel aqueous medium, images were acquired on a fluorescent Olympus BX60 microscope equipped with an Olympus DP74 camera (Spain). The absence of MBP with respect to the total area was assessed in the spinal cord white matter and expressed as a percentage. Meanwhile, MBP immunoreactivity in the PL/IL and M2/M1 was quantified in a region of interest defined in each structure by obtaining the mean intensity after subtracting the background noise from each section (expressed in arbitrary units, AU).

Astrocyte activation in the spinal cord and cortex: As a marker of astrocytes, GFAP immunoreactivity was assessed in the dorsal and ventral horns of the lumbar spinal cord and cortex (4-5 animals per group). Antigen retrieval was first performed to unmask the epitopes, incubating sections in 2N HCl at 37°C (20 min) and then accommodating them for 5 min at room temperature. The sections were then probed for 2 nights at 4ºC with a rabbit antibody against GFAP (1:1000; Dako, USA), the binding of which was detected with a biotinylated donkey anti-rabbit antibody (1:200; Jackson ImmunoResearch Europe, UK), and visualized with an ultra-sensitive ABC peroxidase staining kit (1:1000; Thermo Scientific, Spain) and 3,3'-diaminobenzidine tetrahydrochloride (DAB) [7]. The sections were then mounted on slides, cleared in xylene and coverslipped with DPX, and the images were acquired at the same exposure and illumination settings on an Olympus BX60 microscope equipped with an Olympus DP74 camera. The optical density of GFAP expression was quantified in the grey matter of the dorsal and ventral horns of the spinal cord, and in the PL/IL and M2/M1 cortices, defining a region of interest in each structure and obtaining the mean intensity by subtracting the background noise from each section (expressed in AU).

Microglial activation in the spinal cord and cortex: To evaluate microglial activation, we analyzed the expression of the ionized calcium-binding adapter molecule 1 (Iba1), and that of arginase-1 (Arg1) and the inducible isoform of nitric oxide synthase (iNOS) in these Iba1^{+} cells. Sections (3-5 animals per group) containing the lumbar spinal cord and cortex were probed for 2 nights at 4ºC with a rabbit (1:1000, Wako, USA) or goat antibodies (1:500, Abcam, UK) against

Iba1, combined with a mouse antibody against Arg1 (1:500, BD Biosciences, USA) or a rabbit antibody against iNOS (1:500, Abcam, UK). The distribution of Arg1 and iNOS was visualized with a biotinylated donkey anti-mouse or anti-rabbit IgG (1:200; Jackson ImmunoResearch Europe, UK) in conjunction with Alexa Fluor 488 streptavidin (1:1000; Invitrogen™, USA). Iba1 was detected with an Alexa Fluor 568 conjugated donkey anti-rabbit or anti-goat antibody (1:1000; Invitrogen™, USA). For the quantification of the relative area occupied by Iba1 in the spinal cord and cortex, Alexa Fluor 488 conjugated donkey anti-rabbit (1:1000; Invitrogen™, USA) was used. After washing and mounting in fluoro-gel aqueous medium, the Iba1 and Arg1/Iba1 images were acquired on a fluorescent Olympus BX60 microscope equipped with an Olympus DP74 camera (Spain), while iNOS/Iba1 images were acquired on a Zeiss LSM 880 Confocal microscope (Carl Zeiss Microscopy GmbH, Germany). The relative area occupied by Iba1 in lumbar spinal cord white matter and in the cortex (PL/IL, M2/M1) was quantified. Arg1/Iba1 expression was analyzed as a percentage of the relative Arg1/Iba1 co-localization with respect to total Iba1 area in lumbar spinal cord. The presence of iNOS expression was also evaluated in lumbar spinal cord.

Noradrenergic projections in the spinal cord and cortex: To evaluate the DBH fibers in the dorsal and ventral horns of the lumbar spinal cord and cortex, sections (4-5 animals per group) were probed for 2 nights at 4ºC with a rabbit antibody against DBH (1:500, Abcam, UK). Subsequently, the sections were incubated with biotinylated donkey anti-rabbit antibody (1:200; Jackson ImmunoResearch Europe, UK), and visualized with an ultra-sensitive ABC peroxidase staining kit (1:1000; Thermo Scientific, Spain) and DAB [7]. The sections were then mounted on slides, cleared in xylene and coverslipped with DPX, and the images were acquired at the same exposure and illumination settings on an Olympus BX60 microscope equipped with an Olympus DP74 camera. The optical density of DBH expression was calculated in the grey matter of the dorsal and ventral horns of the spinal cord, and the PL/IL and M2/M1 cortices, defining a region of interest in each region and obtaining the mean intensity by subtracting the background noise from each section (expressed in AU).

DREADD virus expression: To verify the mCherry expression in the LC, and the A5 and A7 noradrenergic areas, sections containing these regions were probed for 2 nights at 4ºC with a rat anti-red fluorescent protein (RFP, 1:500; Chromotek, Germany) and a rabbit anti-DBH (1:500; Abcam, UK) antibodies. The binding of these antibodies was detected with a biotinylated donkey anti-rat antibody (1:200; Jackson ImmunoResearch Europe, UK), which was visualized with Alexa Fluor 568 streptavidin or an Alexa Fluor 488 conjugated donkey anti-rabbit antibody (1:1000; Invitrogen™, USA). Nuclear counterstaining with DAPI (#D9542-10MG, 1:5000, Sigma-Aldrich, USA) was performed and sections were then washed and coverslipped in fluoro-gel aqueous mounting medium and the images were acquired on a Zeiss LSM 880 Confocal

microscope using FAST Airyscan (Carl Zeiss Microscopy GmbH, Germany). The relative mCherry expression in DBH$^+$ neurons of the LC was quantified and expressed in percentage. The selective expression of DREADD was also assessed in the A5 and A7 noradrenergic nuclei [7].

Statistical analysis

The data are represented as the means + standard error of the mean (SEM) and the results were analyzed using GraphPad Prism version 9.4.0 (GraphPad Software, USA), applying a Student's t-test (unpaired, two-tailed), or a one or two-way analysis of variance (ANOVA), with or without repeated measures (RM), followed by a Bonferroni or Dunnett's post hoc test. For RM ANOVA Geisser-Greenhouse correction was applied. For non-parametric data, a Mann-Whitney U or Kruskal-Wallis test was used, followed by Dunn's post hoc test. The differences were considered significant at $p<0.05$ (Tables S2-11). Normality was assessed using the Shapiro-Wilk test and statistical outliers were identified using Grubbs' test. Correlations were determined using a Pearson's or the non-parametric Spearman correlation coefficients.

References

1. INFRAFRONTIER Consortium. INFRAFRONTIER--providing mutant mouse resources as research tools for the international scientific community. Nucleic Acids Res. 2015;43(Database issue):D1171-5.
2. Paxinos G, Franklin KBJ. The Mouse Brain in Stereotaxic Coordinates. 5th Edn ed. Paxinos G, editor. Amsterdam: Elsevier; 2019.
3. Musgrave T, Benson C, Wong G, Browne I, Tenorio G, Rauw G, et al. The MAO inhibitor phenelzine improves functional outcomes in mice with experimental autoimmune encephalomyelitis (EAE). Brain Behav Immun. 2011;25(8):1677-88.
4. An K, Xue MJ, Zhong JY, Yu SN, Lan TS, Qi ZQ, et al. Arsenic trioxide ameliorates experimental autoimmune encephalomyelitis in C57BL/6 mice by inducing CD4. J Neuroinflammation. 2020;17(1):147.
5. Song J, Wu C, Korpos E, Zhang X, Agrawal SM, Wang Y, et al. Focal MMP-2 and MMP-9 activity at the blood-brain barrier promotes chemokine-induced leukocyte migration. Cell Rep. 2015;10(7):1040-54.
6. Yakimov V, Schweiger F, Zhan J, Behrangi N, Horn A, Schmitz C, et al. Continuous cuprizone intoxication allows active experimental autoimmune encephalomyelitis induction in C57BL/6 mice. Histochem Cell Biol. 2019;152(2):119-31.
7. Llorca-Torralba M, Suárez-Pereira I, Bravo L, Camarena-Delgado C, Garcia-Partida JA, Mico JA, et al. Chemogenetic Silencing of the Locus Coeruleus-Basolateral Amygdala Pathway Abolishes Pain-Induced Anxiety and Enhanced Aversive Learning in Rats. Biol Psychiatry. 2019;85(12):1021-35.

Supplementary figures

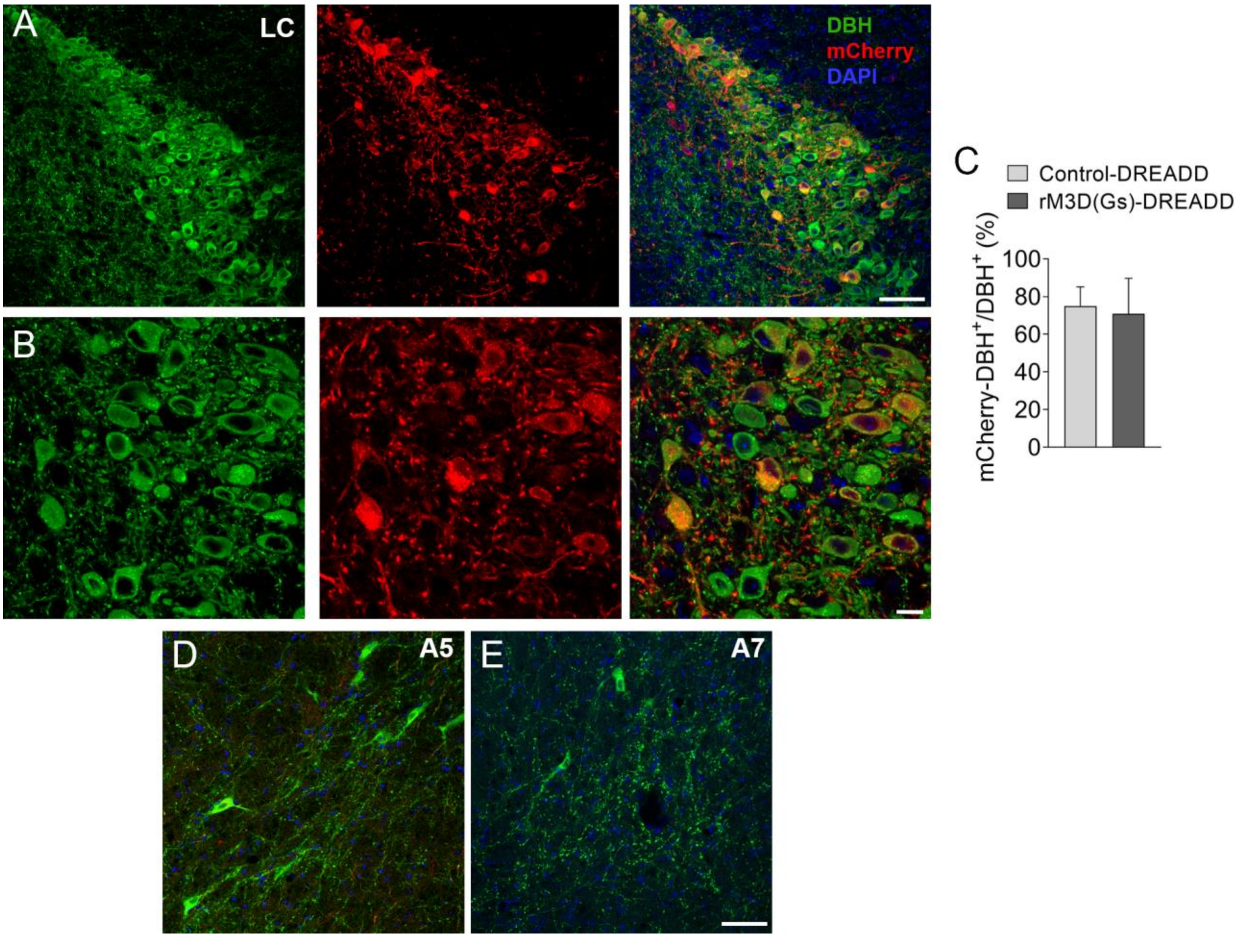

Fig. S1 Validation of mCherry expression in DBH^+ neurons. (A) Representative images showing mCherry (red) expression in DBH^+ (green) neurons in the LC and nuclear counterstaining with DAPI (blue). **(B)** Magnified images showing the mCherry expression in these neurons and **(C)** the graphs indicate the relative mCherry expression in DBH^+ LC neurons. No mCherry expression was observed in **(D)** A5 or **(E)** A7 noradrenergic neurons. The data represent the mean + SEM (n=3). Scale bars: **(A, D, E)**, 50 µm; **(B)**, 20 µm. DBH, dopamine beta-hydroxylase; LC, locus coeruleus

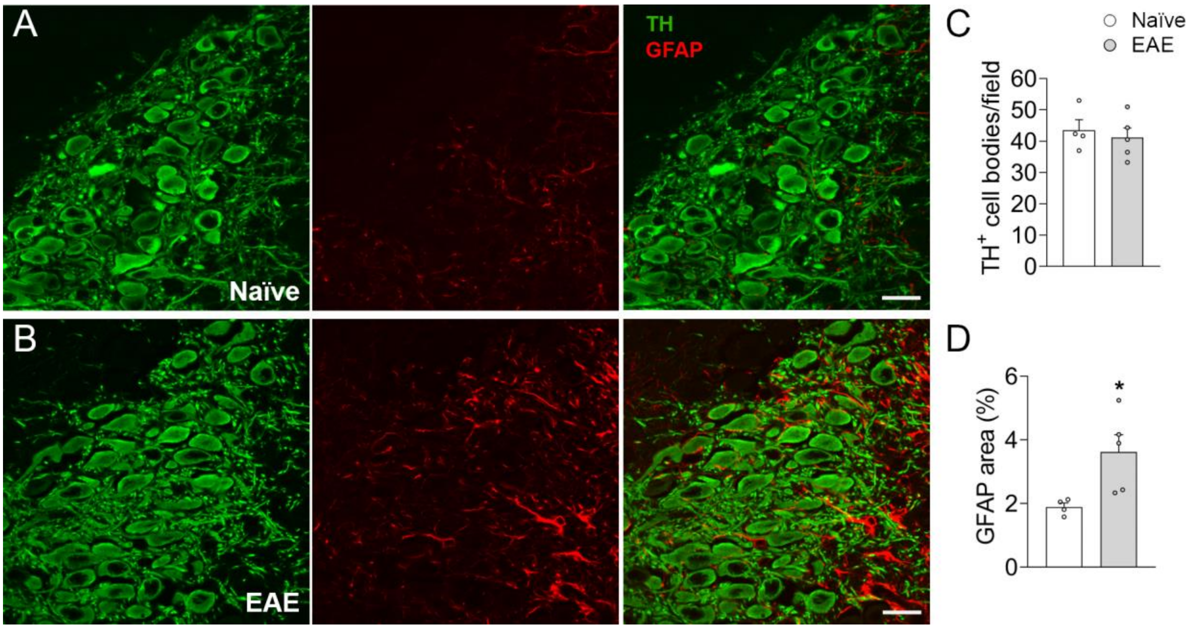

Fig. S2 Assessment of noradrenergic cells and astrocyte status in the LC. Representative images of TH^+ neurons (green) and GFAP (red) staining in the LC of **(A)** naïve and **(B)** EAE animals. **(C)** Quantification of the number of TH^+ neurons and **(D)** the relative area occupied by GFAP in this nucleus. The data represent the mean + SEM and each point corresponds to an individual mouse (n=4-5; Table S9). Scale bars: 20 µm. GFAP, glial fibrillary acidic protein; TH, tyrosine hydroxylase

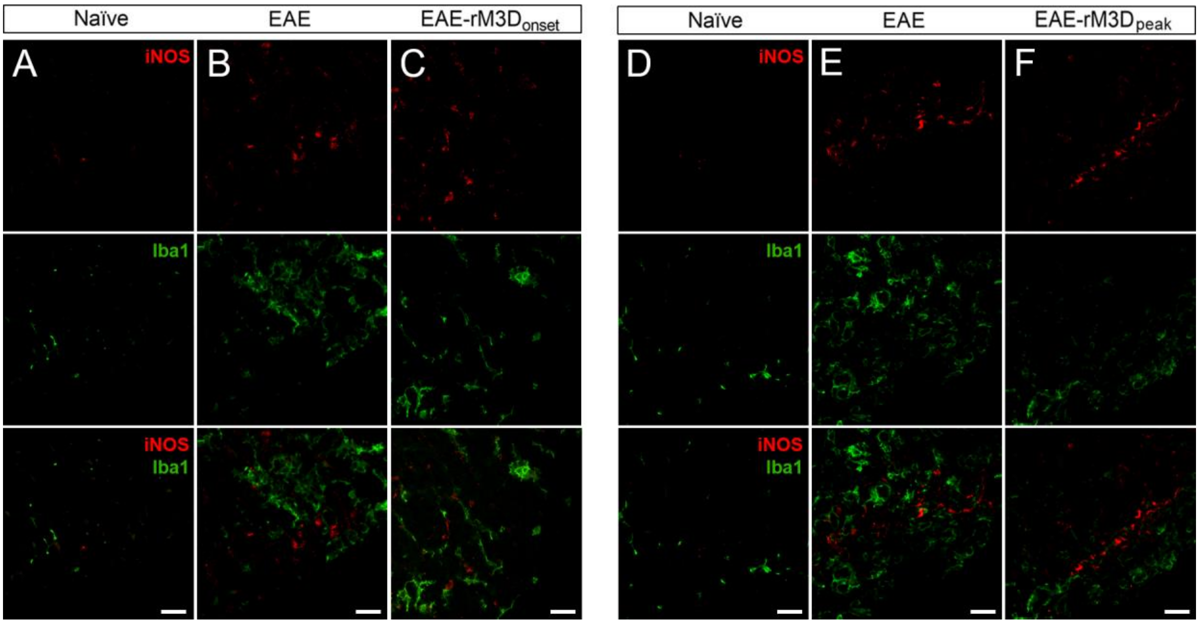

Fig. S3 Evaluation of iNOS and Iba1 co-expression in the lumbar spinal cord. Representative images (n=2) showing iNOS (red) and Iba1 (green) expression in **(A-C)** animals administered clozapine-N-oxide (CNO) from the onset or **(D-F)** from the peak of the motor symptoms. Scale

bars: 50 µm. Iba1, ionized calcium-binding adapter molecule 1; iNOS, inducible isoform of nitric oxide synthase

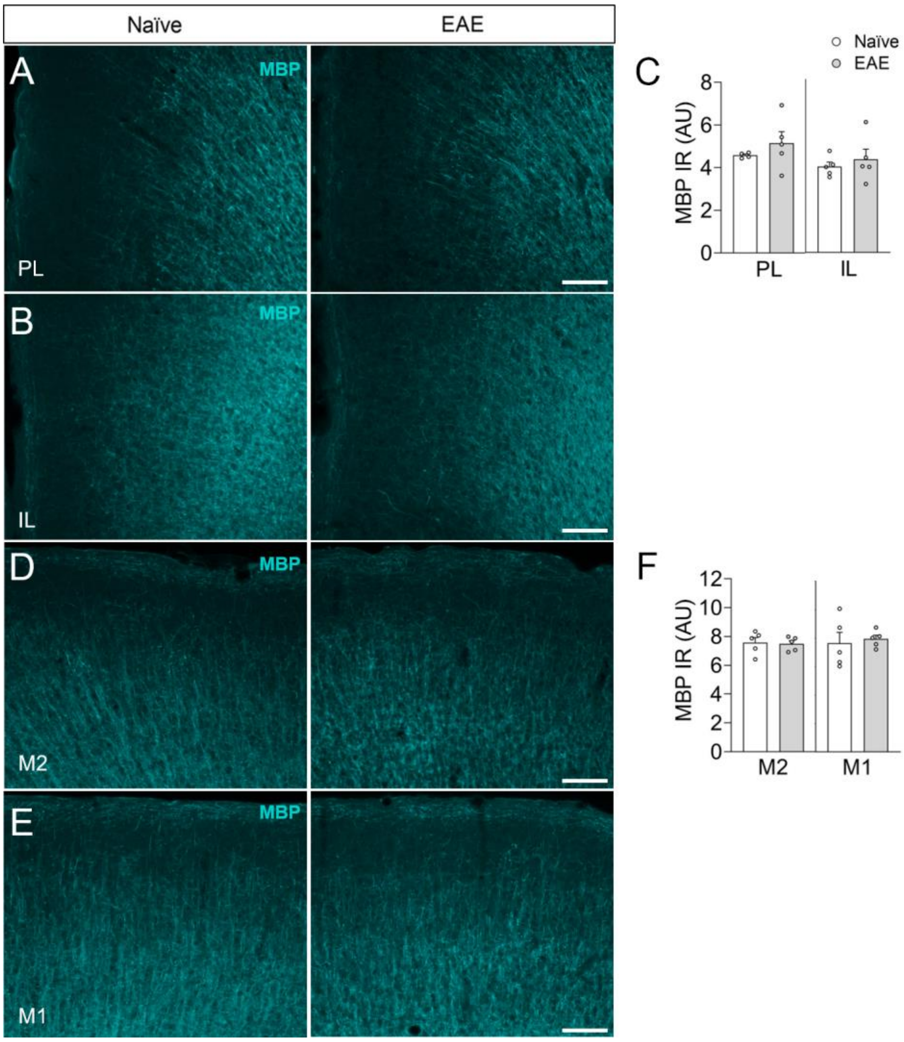

Fig. S4 Cortical demyelination. Representative images showing myelin basic protein (MBP, cyan) expression in the **(A)** PL, **(B)** IL, **(D)** M2 and **(E)** M1 from naïve and EAE animals, quantified as **(C, F)** MBP immunoreactivity (IR) in arbitrary units (AU). The data represent the mean + SEM and each point corresponds to an individual mouse (n=4-5; Table S10). Scale bars: 100 µm. IL, infralimbic cortex; M1, primary motor cortex; M2, secondary motor cortex; PL, prelimbic cortex

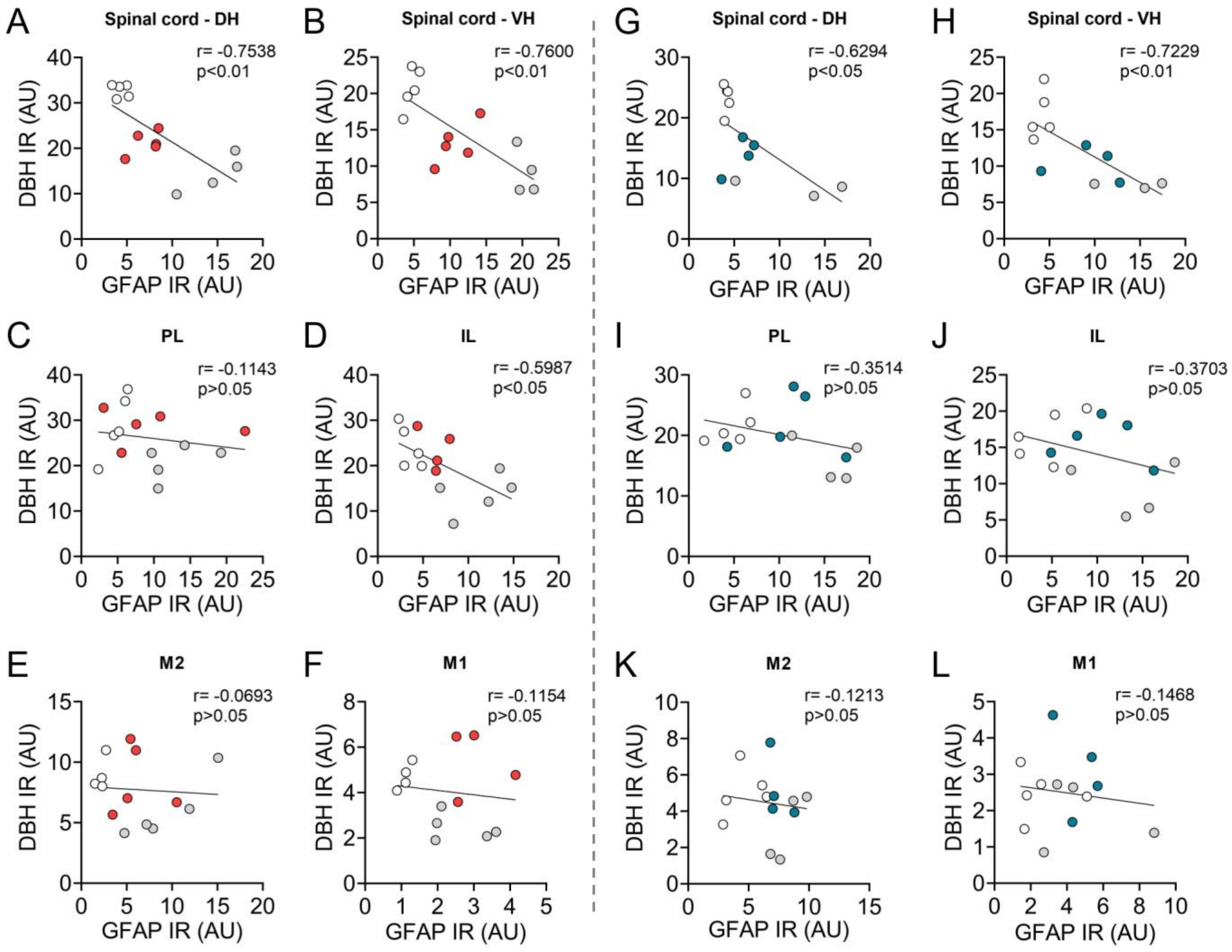

Fig. S5 Correlation between DBH and GFAP expression. DBH (Y-axis) and GFAP (X-axis) immunoreactivity (IR) after chemogenetic LC activation from the onset **(A-F)** or the peak **(G-L)** of the motor symptoms, as assessed in the **(A, G)** dorsal horn (DH) and **(B, H)** ventral horn (VH) of the spinal cord, and in the **(C, I)** PL, **(D, J)** IL, **(E, K)** M2 and **(F, L)** M1 cortices of naïve (white circles), EAE (gray circles), EAE-rM3D$_{onset}$ (red circles) and EAE-rM3D$_{peak}$ (blue circles) mice. Each point corresponds to an individual mouse (n=4-5; Table S11). Pearson or Spearman correlation analysis. DBH, dopamine beta-hydroxylase; GFAP, glial fibrillary acidic protein; IL, infralimbic cortex; M1, primary motor cortex; M2, secondary motor cortex; PL, prelimbic cortex

Table S1. Antibodies used in this study

Primary Antibody	Host Species	Manufacturer	Catalogue Reference	Dilution
Arg1	Mouse	BD Biosciences	BD610708	1:500
DBH	Rabbit	Abcam	ab209487	1:500
GFAP	Mouse	Millipore	MAB360	1:1000
GFAP	Rabbit	Dako	Z0334	1:1000
Iba1	Rabbit	Wako	019-19741	1:1000
Iba1	Goat	Abcam	ab5076	1:500
iNOS	Rabbit	Abcam	ab136918	1:500
MBP	Mouse	Biolegend	808401	1:1000
RFP 5F8 (mCherry)	Rat	Chromotek	5F8	1:500
TH	Sheep	Abcam	ab113	1:1000
**Secondary Antibody**	**Host Species**	**Manufacturer**	**Catalogue Reference**	**Dilution**
anti-mouse biotin	Donkey	Jackson Immunoresearch	715-065-151	1:200
anti-rabbit biotin	Donkey	Jackson Immunoresearch	711-065-152	1:200
anti-rat biotin	Donkey	Jackson Immunoresearch	712-065-153	1:200
Streptavidin-Alexa 488	-------	Invitrogen	S11223	1:1000
Streptavidin-Alexa 568	-------	Invitrogen	S11226	1:1000
anti-rabbit IgG Alexa 488	Donkey	Invitrogen	A21206	1:1000
anti-goat IgG Alexa 568	Donkey	Invitrogen	A11057	1:1000
anti-mouse IgG Alexa 568	Donkey	Invitrogen	A10037	1:1000
anti-rabbit IgG Alexa 568	Donkey	Invitrogen	A10042	1:1000
anti-sheep IgG Alexa 647	Donkey	Invitrogen	A21448	1:1000

Table S2. Summary of statistical analysis of Fig. 1

Shapiro-Wilk normality test *(W, P value, Passed normality test?)*

	Naïve			EAE			EAE-rM3D		
**Clinical score**	---			0.7418<br><0.0001<br>No			0.7890<br>0.0004<br>No		
**AUC (clinical score)**	---			0.9085<br>0.2712<br>Yes			0.8938<br>0.1871<br>Yes		
**Maximum score**	---			0.8482<br>0.0553<br>Yes			0.7485<br>0.0052<br>No		
**Clinical score (%)**					***17 dpi***	***25 dpi***		***17 dpi***	***25 dpi***
	---				0.9272<br>0.4209<br>Yes	0.9331<br>0.4788<br>Yes		0.8335<br>0.0489<br>No	0.7755<br>0.0107<br>No
**Body weight (%)**	0.9395<br>0.4910<br>Yes			0.9259<br>0.3391<br>Yes			0.9550<br>0.7113<br>Yes		
**Body weight (% 27 dpi)**	0.5553<br><0.0001<br>No			0.9085<br>0.3057<br>Yes			0.8185<br>0.0243<br>No		
**Locomotor activity**	0.6045<br><0.0001<br>No			0.7748<br>0.0003<br>No			0.7184<br><0.0001<br>No		
**AUC (locomotor activity)**	0.9507<br>0.7181<br>Yes			0.8821<br>0.1379<br>Yes			0.9523<br>0.6954<br>Yes		
**Time in center**	0.8670<br>0.2870<br>Yes			1.0000<br>0.9969<br>Yes			0.7820<br>0.0723<br>Yes		
**Activity/attention score**	***Onset***	***17 dpi***	***25 dpi***	***Onset***	***17 dpi***	***25 dpi***	***Onset***	***17 dpi***	***25 dpi***
	---	---	---	0.6173<br>0.0002<br>No	0.9285<br>0.4337<br>Yes	0.9107<br>0.2857<br>Yes	0.3898<br><0.0001<br>No	0.8553<br>0.0672<br>Yes	0.8677<br>0.0939<br>Yes
**Sedentary posture**	***Onset***	***17 dpi***	***25 dpi***	***Onset***	***17 dpi***	***25 dpi***	***Onset***	***17 dpi***	***25 dpi***
	0.9636<br>0.8327<br>Yes	0.9277<br>0.5811<br>Yes	0.9530<br>0.7583<br>Yes	0.8869<br>0.3417<br>Yes	0.8864<br>0.3392<br>Yes	0.8878<br>0.3463<br>Yes	0.9630<br>0.8290<br>Yes	0.9262<br>0.5704<br>Yes	0.9746<br>0.9041<br>Yes

Parametric tests

Two-way RM ANOVA *($F_{(df, residual)}$)*

	**Time**	**Group**	**Time x Group**
**Clinical score**	**$F_{(3.009, 54.17)}$ = 66.80*****	**$F_{(1, 18)}$= 8.55****	**$F_{(20, 360)}$= 2.28****
**Clinical score (%)**	$F_{(1, 17)}$= 4.11	**$F_{(1, 17)}$= 5.67***	$F_{(1, 17)}$= 4.11
**Body weight (%)**	**$F_{(4.712, 113.1)}$ = 16.78*****	**$F_{(2, 24)}$= 3.63***	**$F_{(22, 264)}$= 3.54*****
**Locomotor activity**	**$F_{(6.895, 172.4)}$= 22.66*****	**$F_{(2, 25)}$= 91.25*****	**$F_{(40, 500)}$= 2.48*****
**Time in center**	$F_{(1.566, 35.23)}$= 2.82	**$F_{(2, 24)}$= 10.86*****	$F_{(4, 45)}$= 1.20
**Sedentary posture**			
Onset	$F_{(3.544, 88.60)}$= 1.43	**$F_{(2, 25)}$= 5.35***	$F_{(8, 100)}$= 0.39
17 dpi	**$F_{(2.555, 63.88)}$= 11.85*****	**$F_{(2, 25)}$= 26.44*****	**$F_{(8, 100)}$= 6.42*****
25 dpi	$F_{(2.505, 62.63)}$= 2.18	**$F_{(2, 25)}$= 40.25*****	$F_{(8, 100)}$= 1.67

One-way ANOVA *($F_{(df, residual)}$)*

**AUC (locomotor activity)**	**$F_{(2, 25)}$= 100.20*** **		
Unpaired Student's t-test *($t_{(df)}$)*			
**AUC (clinical score)**	**$t_{(18)}$= 2.93** **		
**Non-parametric tests**			
Mann-Whitney test *(P value, Mann-Whitney U)*			
**Maximum score**	**0.0424***<br>**20**		
Kruskal-Wallis test *(P value, Kruskal-Wallis statistic)*			
**Body weight (% 27dpi)**	**0.0101***<br>**9.20**		
**Activity/attention score**	*Onset*	*17 dpi*	*25 dpi*
	0.1603	**0.0001*** **	**<0.0001*** **
	3.66	**17.96**	**20.02**

AUC, area under the curve; df, degrees of freedom; dpi, days post-induction. *p<0.05; **p<0.01; ***p<0.001.

Table S3. Summary of statistical analysis of Fig. 2

Shapiro-Wilk normality test *(W, P value, Passed normality test?)*

	Naïve	EAE	EAE-rM3D
**MBP**	0.9150<br>0.4982<br>Yes	0.9629<br>0.8282<br>Yes	0.9665<br>0.8525<br>Yes
**Infiltration score**	0.7772<br>0.0522<br>Yes	0.9315<br>0.6066<br>Yes	0.9083<br>0.4575<br>Yes
**Cuffs**	0.8768<br>0.2952<br>Yes	0.9791<br>0.9300<br>Yes	0.8794<br>0.3065<br>Yes
**Preclinical cuffs**	---	0.8392<br>0.1627<br>Yes	0.8872<br>0.3433<br>Yes
**GFAP (DH)**	0.9452<br>0.7031<br>Yes	0.8397<br>0.1641<br>Yes	0.8313<br>0.1423<br>Yes
**GFAP (VH)**	0.9932<br>0.9896<br>Yes	0.9319<br>0.6092<br>Yes	0.9421<br>0.6810<br>Yes
**Iba1**	0.8556<br>0.2130<br>Yes	0.9727<br>0.8924<br>Yes	0.8750<br>0.2873<br>Yes
**Arg1**	---	0.9626<br>0.1534<br>Yes	0.9772<br>0.3231<br>Yes

Parametric tests

One-way ANOVA *($F_{(df, residual)}$)*

**MBP**	$\mathbf{F_{(2, 12)} = 25.35}$***
**Infiltration score**	$\mathbf{F_{(2, 12)} = 44.64}$***
**Cuffs**	$\mathbf{F_{(2, 12)} = 37.32}$***
**GFAP (DH)**	$\mathbf{F_{(2, 12)} = 43.01}$***
**GFAP (VH)**	$\mathbf{F_{(2, 12)} = 86.09}$***
**Iba1**	$\mathbf{F_{(2, 12)} = 24.09}$***

Unpaired Student's t-test *($t_{(df)}$)*

**Preclinical cuffs**	$\mathbf{t_{(8)} = 2.33}$*
**Arg1**	$\mathbf{t_{(103)} = 7.09}$***

Arg1, arginase-1; df, degrees of freedom; DH, dorsal horn; GFAP, glial fibrillary acidic protein; Iba1, ionized calcium-binding adapter molecule 1; MBP, myelin basic protein; VH, ventral horn. *p<0.05; **p<0.01; ***p<0.001.

Table S4. Summary of statistical analysis of Fig. 3

Shapiro-Wilk normality test *(W, P value, Passed normality test?)*

	Naïve	EAE	EAE-rM3D
**GFAP (PL)**	0.9074	0.8304	0.8731
	0.4520	0.1400	0.2791
	Yes	Yes	Yes
**GFAP (IL)**	0.8762	0.9197	0.9435
	0.2926	0.5281	0.6758
	Yes	Yes	Yes
**GFAP (M2)**	0.9290	0.9521	0.8640
	0.5885	0.7524	0.2428
	Yes	Yes	Yes
**GFAP (M1)**	0.9428	0.7183	0.9052
	0.6717	0.0186	0.4394
	Yes	No	Yes
**Iba1 (PL)**	0.8312	0.7511	0.7169
	0.1421	0.0305	0.0143
	Yes	No	No
**Iba1 (IL)**	0.8363	0.7963	0.9397
	0.1550	0.0756	0.6640
	Yes	Yes	Yes
**Iba1 (M2)**	0.7781	0.7008	0.8483
	0.0530	0.0098	0.1892
	Yes	No	Yes
**Iba1 (M1)**	0.7411	0.5833	0.9579
	0.0246	0.0004	0.7933
	No	No	Yes

Parametric tests

One-way ANOVA *($F_{(df, residual)}$)*

**GFAP (PL)**	$F_{(2, 12)}$= 3.21
**GFAP (IL)**	**$F_{(2, 11)}$= 14.29*** **
**GFAP (M2)**	**$F_{(2, 11)}$= 6.55***
**Iba1 (IL)**	$F_{(2, 12)}$= 0.78

Non-parametric tests

Kruskal-Wallis test *(P value, Kruskal-Wallis statistic)*

**GFAP (M1)**	**0.0009***<br>9.17**
**Iba1 (PL)**	0.4969<br>1.52
**Iba1 (M2)**	0.2231<br>3.12
**Iba1 (M1)**	0.1646<br>3.66

df, degrees of freedom; GFAP, glial fibrillary acidic protein; Iba1, ionized calcium-binding adapter molecule 1; IL, infralimbic cortex MBP, myelin basic protein; M1, primary motor cortex; M2, secondary motor cortex; PL, prelimbic cortex. *p<0.05; **p<0.01; ***p<0.001.

Table S5. Summary of statistical analysis of Fig. 4

Shapiro-Wilk normality test** (W, P value, Passed normality test?) **for DBH

DBH	Naïve	EAE	EAE-rM3D
**Spinal cord - DH**	0.8032	0.9830	0.9849
	0.0860	0.9194	0.9592
	Yes	Yes	Yes
**Spinal cord - VH**	0.9497	0.8564	0.9822
	0.7351	0.2476	0.9461
	Yes	Yes	Yes
**PL**	0.9541	0.8907	0.9557
	0.7667	0.3606	0.7775
	Yes	Yes	Yes
**IL**	0.8785	0.9621	0.9782
	0.3026	0.8228	0.9250
	Yes	Yes	Yes
**M2**	0.8481	0.7881	0.8605
	0.1886	0.0646	0.2300
	Yes	Yes	Yes
**M1**	0.9722	0.8098	0.9121
	0.8893	0.0972	0.4803
	Yes	Yes	Yes

Parametric tests

One-way ANOVA** ($F_{(df, residual)}$) **for DBH

**Spinal cord - DH**	**$F_{(2, 11)}$= 48.84*** **
**Spinal cord - VH**	**$F_{(2, 11)}$= 18.22*** **
**PL**	**$F_{(2, 12)}$= 4.08***
**IL**	**$F_{(2, 12)}$= 5.98***
**M2**	$F_{(2, 12)}$= 2.92
**M1**	$F_{(2, 12)}$= 1.68

DBH, dopamine beta-hydroxylase; df, degrees of freedom; DH, dorsal horn; GFAP, glial fibrillary acidic protein; IL, infralimbic cortex; M1, primary motor cortex; M2, secondary motor cortex; PL, prelimbic cortex; VH, ventral horn. *p<0.05; **p<0.01; ***p<0.001.

Table S6. Summary of statistical analysis of Fig. 5

Shapiro-Wilk normality test *(W, P value, Passed normality test?)*

	Naïve			EAE			EAE-rM3D		
**Clinical score**					0.7851			0.7505	
		---			0.0002			<0.0001	
					No			No	
**AUC (clinical score)**					0.9237			0.9335	
		---			0.4986			0.5150	
					Yes			Yes	
**Maximum score**					0.8335			0.7765	
		---			0.0489			0.0238	
					No			No	
**Clinical score (%)**				***17 dpi***		***25 dpi***	***17 dpi***		***25 dpi***
				0.6827		0.9366	0.9131		0.8096
		---		0.004		0.6085	0.3379		0.0263
				No		Yes	Yes		No
**Body weight (%)**		0.9146			0.8144			0.9162	
		0.2118			0.0101			0.2227	
		Yes			No			Yes	
**Body weight (% 29 dpi)**		0.9808			0.9099			0.9445	
		0.9693			0.3955			0.6296	
		Yes			Yes			Yes	
**Locomotor activity**		0.7472			0.7957			0.8333	
		<0.0001			0.0003			0.0014	
		No			No			No	
**AUC (locomotor activity)**		0.9342			0.9397			0.9350	
		0.4906			0.6363			0.5300	
		Yes			Yes			Yes	
**Time in center**		0.9395			0.9025			0.9957	
		0.5253			0.3935			0.8746	
		Yes			Yes			Yes	
**Activity/attention score**	***Onset***	***17 dpi***	***25 dpi***	***Onset***	***17 dpi***	***25 dpi***	***Onset***	***17 dpi***	***25 dpi***
				0.6004	0.9442	0.8528	0.5659	0.8198	0.8232
	---	---	---	0.0003	0.6766	0.1305	<0.0001	0.0343	0.0375
				No	Yes	Yes	No	No	No
**Sedentary posture**	***Onset***	***17 dpi***	***25 dpi***	***Onset***	***17 dpi***	***25 dpi***	***Onset***	***17 dpi***	***25 dpi***
	0.9556	0.9141	0.9386	0.7557	0.8285	0.7369	0.9250	0.7691	0.8589
	0.7773	0.4925	0.6557	0.0336	0.1356	0.0224	0.5629	0.0443	0.2242
	Yes	Yes	Yes	No	Yes	No	Yes	No	Yes

Parametric tests

Two-way RM ANOVA *($F_{(df, residual)}$)*

	**Time**	**Group**	**Time x Group**
**Clinical score**	$F_{(3.264, 45.70)}$ = 74.37***	$F_{(1, 14)}$= 0.06	$F_{(22, 308)}$= 0.89
**Clinical score (%)**	$F_{(1, 13)}$= 24.02***	$F_{(1, 14)}$= 0.59	$F_{(1, 13)}$= 1.73
**Body weight (%)**	$F_{(4.437, 102.0)}$= 34.15***	$F_{(2, 23)}$= 13.91***	$F_{(24, 276)}$= 5.42***
**Locomotor activity**	$F_{(6.608, 152.0)}$= 28.60***	$F_{(2, 23)}$= 51.77***	$F_{(44, 506)}$= 2.03***
**Time in center**	$F_{(1.632, 31.82)}$= 5.02*	$F_{(2, 25)}$= 3.48*	$F_{(4, 39)}$= 0.88
**Sedentary posture**			
Onset	$F_{(2.604, 57.30)}$= 5.82**	$F_{(2, 22)}$= 8.08**	$F_{(8, 88)}$= 2.66*
17 dpi	$F_{(2.390, 54.98)}$= 12.29***	$F_{(2, 23)}$= 10.25***	$F_{(8, 92)}$= 3.61**
25 dpi	$F_{(3.264, 75.08)}$= 2.35	$F_{(2, 23)}$= 25.34***	$F_{(8, 92)}$= 3.95***

One-way ANOVA *($F_{(df, residual)}$)*

**Body weight (% 27dpi)**	$F_{(2, 23)}$= 10.47***

**AUC (locomotor activity)**	**$F_{(2, 23)}$= 47.09*** **		
Unpaired Student's t-test *($t_{(df)}$)*			
**AUC (clinical score)**	$t_{(14)}$= 1.14		
**Non-parametric tests**			
Mann-Whitney test *(P value, Mann-Whitney U)*			
**Maximum score**	>0.99<br>31.5		
Kruskal-Wallis test *(P value, Kruskal-Wallis statistic)*			
**Activity/attention score**	*Onset*	*17 dpi*	*25 dpi*
	0.2142	**0.0004*** **	**<0.0001*** **
	3.08	**15.81**	**18.55**

AUC, area under the curve; df, degrees of freedom; dpi, days post-induction. *p<0.05; **p<0.01; ***p<0.001.

Table S7. Summary of statistical analysis of Fig. 6

***Shapiro-Wilk normality test** (W, P value, Passed normality test?)*

	Naïve	EAE	EAE-rM3D
**MBP**	0.9131	0.9128	0.9593
	0.4863	0.4848	0.8029
	Yes	Yes	Yes
**Infiltration score**	0.9022	0.8955	0.8824
	0.4221	0.3855	0.3204
	Yes	Yes	Yes
**Perivascular cuffs**	0.9250	0.9568	0.9267
	0.5624	0.7859	0.5739
	Yes	Yes	Yes
**Preclinical cuffs**		0.8970	0.9832
	---	0.3936	0.9512
		Yes	Yes
**GFAP (DH)**	0.9372	0.9639	0.9617
	0.6461	0.8037	0.8197
	Yes	Yes	Yes
**GFAP (VH)**	0.8782	0.8641	0.9669
	0.3013	0.2750	0.8552
	Yes	Yes	Yes
**Iba1**	0.9723	0.8268	0.8327
	0.8895	0.1316	0.1458
	Yes	Yes	Yes
**Arg1**		0.9412	0.9748
	---	0.0238	0.2476
		No	Yes

Parametric tests

***One-way ANOVA** ($F_{(df,\ residual)}$)*

**MBP**	**$F_{(2, 12)}$= 51.79*** **
**Infiltration score**	**$F_{(2, 12)}$= 134.00*** **
**Perivascular cuffs**	**$F_{(2, 12)}$= 22.61*** **
**GFAP (DH)**	**$F_{(2, 11)}$= 9.43** **
**GFAP (VH)**	**$F_{(2, 11)}$= 16.45*** **
**Iba1**	**$F_{(2, 12)}$= 23.22*** **

***Unpaired Student's t-test** ($t_{(df)}$)*

**Preclinical cuffs**	$t_{(8)}$= 2.01

Non-parametric tests

***Mann-Whitney test** (P value, Mann-Whitney U)*

**Arg1**	0.1695<br>1137

Arg1, arginase-1; df, degrees of freedom; DH, dorsal horn; GFAP, glial fibrillary acidic protein; Iba1, ionized calcium-binding adapter molecule 1; MBP, myelin basic protein; VH, ventral horn. *p<0.05; **p<0.01; ***p<0.001.

Table S8. Summary of statistical analysis of Fig. 7

Shapiro-Wilk normality test *(W, P value, Passed normality test?)*

	Naïve	EAE	EAE-rM3D
**GFAP (PL)**	0.9088	0.9073	0.9737
	0.4607	0.4517	0.8982
	Yes	Yes	Yes
**GFAP (IL)**	0.8936	0.9042	0.9885
	0.3758	0.4337	0.9744
	Yes	Yes	Yes
**GFAP (M2)**	0.8795	0.9399	0.8978
	0.3069	0.6652	0.3978
	Yes	Yes	Yes
**GFAP (M1)**	0.7737	0.8785	0.8733
	0.0486	0.3027	0.2801
	No	Yes	Yes
**DBH (Spinal cord - DH)**	0.9004	0.8698	0.9400
	0.4122	0.2968	0.6542
	Yes	Yes	Yes
**DBH (Spinal cord - VH)**	0.9092	0.9529	0.9779
	0.4630	0.7342	0.8895
	Yes	Yes	Yes
**DBH (PL)**	0.8303	0.8511	0.8863
	0.1398	0.2296	0.3388
	Yes	Yes	Yes
**DBH (IL)**	0.9400	0.8680	0.9756
	0.6662	0.2898	0.9099
	Yes	Yes	Yes
**DBH (M2)**	0.9699	0.7969	0.8006
	0.8743	0.0968	0.1032
	Yes	Yes	Yes
**DBH (M1)**	0.9597	0.8627	0.9979
	0.8056	0.2701	0.9931
	Yes	Yes	Yes

Parametric tests

One-way ANOVA *($F_{(df, residual)}$)*

**GFAP (PL)**	**$F_{(2, 12)}$= 10.69****
**GFAP (IL)**	**$F_{(2, 12)}$= 4.42***
**GFAP (M2)**	**$F_{(2, 12)}$= 5.64***
**DBH (Spinal cord - DH)**	**$F_{(2, 10)}$= 48.53****
**DBH (Spinal cord - VH)**	**$F_{(2, 10)}$= 18.01*****
**DBH (PL)**	$F_{(2, 11)}$= 2.71
**DBH (IL)**	**$F_{(2, 11)}$= 6.19***
**DBH (M2)**	$F_{(2, 10)}$= 2.04
**DBH (M1)**	$F_{(2, 10)}$= 1.64

Non-parametric tests

Kruskal-Wallis test *(P value, Kruskal-Wallis statistic)*

**GFAP (M1)**	0.0626<br>5.42

DBH, dopamine beta-hydroxylase; df, degrees of freedom; DH, dorsal horn; GFAP, glial fibrillary acidic protein; IL, infralimbic cortex; M1, primary motor cortex; M2, secondary motor cortex; PL, prelimbic cortex; VH, ventral horn. *p<0.05; **p<0.01; ***p<0.001.

Table S9. Summary of statistical analysis of Fig. S2

Shapiro-Wilk normality test *(W, P value, Passed normality test?)*

	Naïve	EAE
**TH (LC)**	0.8990	0.9778
	0.4263	0.9228
	Yes	Yes
**GFAP (LC)**	0.9391	0.9108
	0.6489	0.4722
	Yes	Yes

Parametric tests

Unpaired Student's t-test *($t_{(df)}$)*

**TH (LC)**	$t_{(7)}$= 0.5184
**GFAP (LC)**	**$t_{(7)}$= 2.72***

df, degrees of freedom; GFAP, glial fibrillary acidic protein; LC, locus coeruleus; TH, tyrosine hydroxylase.

Table S10. Summary of statistical analysis of Fig. S4

Shapiro-Wilk normality test *(W, P value, Passed normality test?)* ***for MBP***

MBP	Naïve	EAE
**PL**	0.9364	0.9759
	0.6326	0.9115
	Yes	Yes
**IL**	0.9386	0.8940
	0.6561	0.3777
	Yes	Yes
**M2**	0.9261	0.8850
	0.5699	0.3326
	Yes	Yes
**M1**	0.9013	0.9821
	0.4173	0.9453
	Yes	Yes

Parametric tests

Unpaired Student's t-test *($t_{(df)}$)* ***for MBP***

**PL**	$t_{(7)}$= 0.94
**IL**	$t_{(8)}$= 0.65
**M2**	$t_{(8)}$= 0.21
**M1**	$t_{(8)}$= 0.37

df, degrees of freedom; IL, infralimbic cortex; M1, primary motor cortex; M2, secondary motor cortex; PL, prelimbic cortex.

Table S11. Summary of statistical analysis of Fig. S5

Shapiro-Wilk normality test** (W, P value, Passed normality test?) **for GFAP and DBH expression

ONSET		PEAK	
**GFAP (Spinal cord - DH)**	0.8584<br>0.0229<br>No	**GFAP (Spinal cord - DH)**	0.7570<br>0.0015<br>No
**DBH (Spinal cord - VH)**	0.9239<br>0.2501<br>Yes	**DBH (Spinal cord - VH)**	0.8975<br>0.1236<br>Yes
**GFAP (Spinal cord - VH)**	0.8942<br>0.0776<br>Yes	**GFAP (Spinal cord - VH)**	0.8869<br>0.0730<br>Yes
**DBH (Spinal cord - VH)**	0.9514<br>0.5829<br>Yes	**DBH (Spinal cord - VH)**	0.8936<br>0.1092<br>Yes
**GFAP (PL)**	0.8742<br>0.089<br>No	**GFAP (PL)**	0.9433<br>0.4263<br>Yes
**DBH (PL)**	0.9841<br>0.9899<br>Yes	**DBH (PL)**	0.9158<br>0.1915<br>Yes
**GFAP (IL)**	0.9378<br>0.3904<br>Yes	**GFAP (IL)**	0.9397<br>0.3792<br>Yes
**DBH (IL)**	0.9750<br>0.9237<br>Yes	**DBH (IL)**	0.9567<br>0.6682<br>Yes
**GFAP (M2)**	0.9117<br>0.1669<br>Yes	**GFAP (M2)**	0.9427<br>0.4182<br>Yes
**DBH (M2)**	0.9530<br>0.5734<br>Yes	**DBH (M2)**	0.9363<br>0.4108<br>Yes
**GFAP (M1)**	0.9518<br>0.6263<br>Yes	**GFAP (M1)**	0.9242<br>0.2230<br>Yes
**DBH (M1)**	0.9227<br>0.2120<br>Yes	**DBH (M1)**	0.9590<br>0.7384<br>Yes

Parametric tests

Pearson correlation** (r, P value) **for GFAP and DBH expression

ONSET		PEAK	
**Spinal cord - VH**	**-0.7600**<br>**0.0016****	**Spinal cord - VH**	**-0.7229**<br>**0.0079****
**PL**	-0.0893<br>0.7532	**PL**	-0.4443<br>0.1114
**IL**	**-0.5891**<br>**0.0267***	**IL**	-0.3883<br>0.1700
**M2**	-0.0693<br>0.8138	**M2**	-0.1213<br>0.6930
**M1**	-0.1270<br>0.6792	**M1**	-0.1468<br>0.6322

Non-parametric tests

Spearman correlation** (r, P value) **for GFAP-DBH expression

ONSET		PEAK	
**Spinal cord - DH**	**-0.7538**<br>**0.0027****	**Spinal cord - DH**	**-0.6294**<br>**0.0323***

DBH, dopamine beta-hydroxylase; DH, dorsal horn; GFAP, glial fibrillary acidic protein; IL, infralimbic cortex; M1, primary motor cortex; M2, secondary motor cortex; PL, prelimbic cortex; VH, ventral horn. *$p<0.05$; **$p<0.01$.
